# Population, land use and economic exposure estimates for Europe at 100 m resolution from 1870 to 2020

**DOI:** 10.1038/s41597-023-02282-0

**Published:** 2023-06-08

**Authors:** Dominik Paprotny, Matthias Mengel

**Affiliations:** Potsdam Institute for Climate Impact Research (PIK), Member of the Leibniz Association, P.O. Box 60 12 03, 14412 Potsdam, Germany

**Keywords:** Natural hazards, Geography, Economics

## Abstract

Understanding the influence of climate change on past extreme weather impacts is a vital research task. However, the effects of climate change are obscured in the observed impact data series due to the rapid evolution of the social and economic circumstances in which the events occurred. The HANZE v2.0 (Historical Analysis of Natural HaZards in Europe) dataset presented in this study quantifies the evolution of key socioeconomic drivers in Europe since 1870, namely land use, population, economic activity and assets. It consists of algorithms to reallocate baseline (2011) land use and population for any given year based on a large collection of historical subnational- and national-level statistics, and then disaggregate data on production and tangible assets by economic sector into a high-resolution grid. Raster datasets generated by the model enable reconstructing exposure within the footprint of any extreme event both at the time of occurrence and anytime between 1870 and 2020. This allows the separation of the effects of climate change from the effects of exposure change.

## Background & Summary

Global mean temperature has surpassed 1 °C warming compared to pre-industrial times. There is growing research that quantifies the effects of the changing climate on the world’s natural, managed and human systems^1,2^. However, less quantification is available for systems with strong non-climatic drivers of change^3^. Case studies have indicated strong influence of additional drivers especially for floods, where the uncertainty of the present risk is already high. For example, flood risk in the Rhine basin was found to be least sensitive to change in atmospheric forcing, but more to changes in reservoir capacity, dike height, land use, asset value or private precautionary measures^4^. Vousdoukas *et al*.^5^ has shown that flood protection was the biggest source of uncertainty in coastal flood risk assessments in test sites in the Iberian Peninsula. Estimates on the value of assets in a given location (exposure) and flood vulnerability functions, which indicate the share of assets that are lost at a given intensity of flood, vary drastically between countries^6–9^. Windstorm damage in Europe was shown not to increase after correcting for exposure increase^10^ with attribution being complicated by contrasting trends in hazard^11^ and very high uncertainty on vulnerability functions^12^. Finally, only a small fraction of wildfires in Europe are caused by natural sources, making the human factors fundamental in understanding the frequency of those disasters^13^.

Many studies indicated no upwards trend in natural hazard direct economic loss in Europe, USA or Australia when corrected for growth in exposure^14–18^. Quantifying changes in exposure, such as land use type, population, economic output, value of assets, and the uncertainty of it is vital not only due to its large direct influence on the observed impacts, but also indirect effects. In case of floods, high-exposure areas tend to be better protected^19^ and less vulnerable^20^, while land-use can locally modulate river discharge stronger than climate change^21^.

Available historic reconstructions of exposure have limited utility for climate change attribution in a long perspective, either due to low resolution, limited spatial coverage or covering only a particular component of exposure. For example, HILDA^22–24^ includes only highly aggregated land cover for the European Union countries, though with a high 1 km resolution covering years 1900 to 2010. The global dataset HYDE^25^ spans from years 10,000 BC to 2017 AD for both land-use and population, but has a resolution of only 5 arc-minutes (9 km on the equator). HYDE is applied extensively in both global climate and climate impact modelling, including ISIMIP^26^. Based on HYDE, a GDP disaggregation was also created^27^ and used e.g. in the global flood attribution study by Sauer *et al*.^28^. Analysing flood and wildfire risk in particular require very high resolution of exposure data because they are highly local phenomena. Yet, high-resolution population data is available at best for a few timesteps per dataset, going back no further than 1975^29^. Disaggregation of economic data is mostly limited to a single predictor of economic activity, such as population density^30^ or night-time lights^31^.

HANZE (Historical Analysis of Natural Hazards in Europe) dataset, released in 2017^32^, was the first comprehensive exposure dataset with resolution matching pan-European flood hazard maps, namely 100 m^33,34^, covering the years 1870 to 2015 with a short-term projection to 2020. It was designed specifically to enable the analysis of exposure and land-use change within flood footprints of known historical floods and was used in such role in various follow-up studies^16,35,36^. Here, we present a revised and expanded exposure dataset HANZE v2.0, which incorporates many improvements (Table 1). The core of the dataset is a set of high-resolution grids of land use, population, gross domestic product (GDP), fixed asset value and soil sealing degree for 42 countries between 1870 and 2020. It is supplemented by a large input database of subnational historical statistics. Further, the dataset is the output of a Python toolbox that enables reproducing the data in full, visualizing it and carry out further analyses (see ‘Usage Notes’).

**Table 1:** Comparison between releases of HANZE dataset.

Aspect	HANZE v1.0 (Paprotny *et al*.^32^)	HANZE v2.0 (this study)
Spatial coverage	36 countries and territories	42 countries and territories
Temporal coverage	1870–2015 and projection for 2020	1870–2020
Spatial resolution	100 m	100 m
Temporal resolution	10-yearly (1870–1970)	10-yearly (1870–1950)
	5-yearly (1970–2020)	5-yearly (1950–2000)
		Annual (2000–2020)
Output exposure rasters	Land cover/use, population, GDP, fixed assets	Land cover/use, population, GDP, fixed assets, soil sealing degree
Validated components	Population change only	Population disaggregation, population, & land use and soil sealing degree change
Uncertainty quantification in the modelling chain	None	Yes, for sub-regional population change and agricultural land-use
Probabilistic outputs	No	Population, GDP, fixed asset value per defined hazard zones
Implementation language	MATLAB 2016a, Python 2.7	Python 3.9
Code availability	Not published	Openly available
Input data availability	Partial	All data published
Flood impact data	1564 events (1870–2016)	Not included (updated data will be added in the future)

The exposure dataset was created with a combination of statistical and rule-based methods. In parts of the model, we included probabilistic methods to quantify the uncertainty, utilizing copulas for modelling sub-regional population changes and a Bayesian Network for agricultural land transitions. The dataset is focused on high-exposure areas that are most relevant for research on the social and economic impacts of disasters. Therefore, changes to some of the less important land use classes, particularly natural areas, were not modelled. Also, large variations in availability and resolution of historical data for different countries directly influence the accuracy of the gridded reconstruction of past exposure. The high resolution of the dataset is provided to enable quick application to hazards such as floods which require such detailed information. Due to the general lack of comparable data at the same resolution, only partial validation can be performed.

## Methods

### Overview

**Fig. 1:**
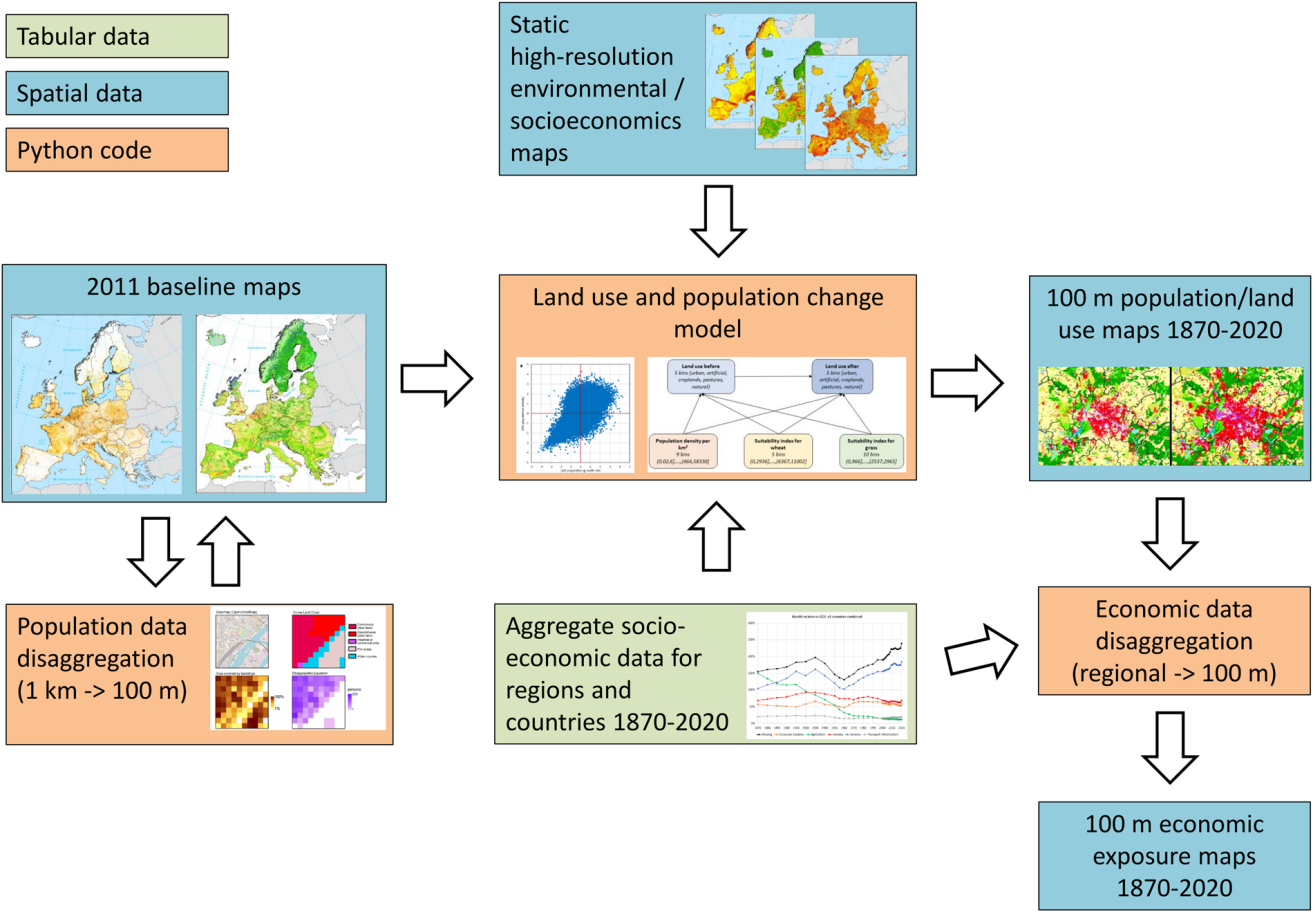
HANZE v2.0 workflow. All input data and Python code needed to reproduce this workflow are publicly available.

HANZE v2.0 is a dataset of historical exposure generated through operations on a large number of input raster data. A summary of the workflow to compute the dataset is presented in Fig. 1. The starting point is a set of high-resolution rasters with data on population and land cover/use for a specific benchmark year. Those “baseline” datasets were created from 100-m resolution data, except population, which was disaggregated from a 1-km resolution (see subsection ‘Baseline datasets’). The model modifies the baseline raster datasets by redistributing land cover/use and population until they match the total population and area of different land-use classes defined per subnational administrative unit for each timestep. For each such unit, we collected aggregate socioeconomic statistics (‘Input socioeconomic data’). Various land-use types (urban, industrial, agriculture etc.) are modelled using different methods and several auxiliary static raster datasets (‘Population and land-use model’). Based on land-use changes, the soil sealing dataset is modified. Finally, the model disaggregates statistical data on gross domestic product (GDP) and fixed asset stock into a 100-m grid, based on the distribution of population and different land-use types (‘Economic data disaggregation’). The model is applied using data covering 42 countries and territories over the period 1870–2020 (see Supplementary Fig. S1).

### Baseline datasets

Four baseline datasets are a set of raster layers covering the study area, closely aligned in the temporal dimension, converted from their native resolutions to a 100 m grid and adjusted to a single land mask based on Corine Land Cover (Table 2).

**Table 2:** List of input baseline geospatial datasets used by the model.

Dataset type	Dataset name	Provider	Native resolution	Timestamp
Land cover/use	Corine Land Cover	Copernicus LMS	100 m	~2012
Soil sealing	Imperviousness Density	Copernicus LMS	20 m	~2012
Population	GEOSTAT	Eurostat	1 km	~2011
Administrative boundaries	NUTS regions	Own work based on open data (see Table 5)	Vector dataset	~2010

#### Land cover/use, soil sealing degree

The baseline land cover/use is taken from Corine Land Cover (CLC) 2012, version 20u1 (https://land.copernicus.eu/pan-european/corine-land-cover/clc-2012), with open sea and some transitional waters removed. The CLC 2012 dataset was created, in general, by manual classification of land cover patches from satellite imagery collected during 2011–2012. The inventory consists of 44 classes and the minimum size of areal phenomena captured is 25 hectares. For linear features (roads, railways, rivers etc.), a minimum width of 100 m is used. The CLC dataset doesn’t cover Andorra, hence a compilation of land use data from other sources was carried over from HANZE v1.0 for Andorra (see section 2.1 in Paprotny *et al*.^32^).

In many places natural land cover was replaced by artificial impervious surfaces. This impermeable cover has a significant impact on hydrological properties of a given area and, consequently, on flood frequency and intensity. It is also an important predictor of asset distribution. The baseline soil sealing dataset in our model is the Imperviousness Density 2012 dataset from Copernicus Land Monitoring Service (https://land.copernicus.eu/pan-european/high-resolution-layers/imperviousness/status-maps/2012). It was created by algorithmic classification of high-resolution satellite images with a calibrated normalised difference vegetation index (NDVI). The native resolution of the dataset is 20 m, but we aggregated it to 100 m for consistency with the land cover dataset.

#### Population

The baseline population dataset is based on the GEOSTAT population grid for the year 2011, version 2.0.1 (https://ec.europa.eu/eurostat/web/gisco/geodata/reference-data/population-distribution-demography/geostat). This dataset has a 1 km resolution and is based on the results of the 2011 round of European population censuses. 95% of the population in the dataset is the actual enumerated and georeferenced during the census, with the remaining population disaggregated from detailed subnational census returns by the European Commission Joint Research Centre. As in HANZE v1.0, we disaggregate this dataset further to a 100 m grid by combining methods “M1” and “M3” described in Batista e Silva *et al*.^37^. “M1” denotes the ‘limiting variable method’ used in cartography for creating dasymetric maps of population density. Briefly, it ranks land use classes according t their average population density, then redistributes population above a land use-specific threshold from less-dense to more-dense classes. The procedure is an iterative algorithm applied separately for each 1 km grid cell. This procedure is as follows:

- Firstly, uniform population density is assigned for each land use class in a 1 km grid cell:
  1 $${Y}_{LG}^{0}={Y}_{G}=\frac{{X}_{G}}{{S}_{G}}$$
  where ${Y}_{LG}^{0}$ is the population density for land use $L\in \{1,\ldots ,n\}$ in grid cell *G* at step 0, *Y*_*G*_ is the population density in the grid cell, i.e. population number *X*_*G*_ divided by area *S*_*G*_.
- A population density threshold *T*_*L*_ is defined for each one of *n* land use classes.
- Land use classes are ranked and the subindex *L* is renumbered from lowest to highest population density, i.e. *L* = 1 denotes the least densely population land use class in the grid cell
- Proceeding in order starting with *L* = 1, in step *L* the density attributed to class *L* in the previous step is modified if it is above the threshold, i.e. if ${Y}_{LG}^{L-1} > {T}_{L}$. That creates a surplus population ${U}_{LG}^{L}$:
  2 $${U}_{LG}^{L}={S}_{LG}\times \left({Y}_{LG}^{L-1}-{T}_{L}\right)$$
- Surplus is then redistributed among the remaining land use classes *M*, hence:

3 $${Y}_{LG}^{L}={T}_{L}$$

4 $${Y}_{MG}^{L}={Y}_{MG}^{L-1}+\frac{{U}_{LG}^{L}}{\sum {S}_{MG}},M > L$$

- If after completing all iterations there is still surplus population, i.e. if ${X}_{G} > \sum {T}_{L}{S}_{LG}$, it is redistributed proportionally to the threshold:

5 $${Y}_{LG}=\frac{{T}_{L}{X}_{G}}{\sum {T}_{L}{S}_{LG}}$$

The crucial aspect of this method is defining the thresholds *T*_*L*_. Here, we use thresholds as suggested by Eicher and Brewer^38^, i.e. the 70^th^ percentile of the population density of grid cells for which only one land use class was reported in our baseline land use dataset. Such “pure” cells constituted around 5% of all population grid cells. Gallego *et al*.^39^ have shown that a different definition of thresholds works slightly better for Europe; however, the authors used population data by communes, which are not used here, and which their method would require in combination with gridded data. The final thresholds *T*_*L*_ are shown in Table 3. For artificial surfaces other than urban fabric, the CLC classes were merged for the threshold calculation, as very few, if any, “pure” cells could be found for each of those classes. Also, for all areas covered by wetlands, water, sand, glaciers, bare rocks or burnt vegetation the threshold was set at 0, as those terrains are in principle uninhabitable.

**Table 3:** Thresholds for population disaggregation algorithm *T*_*L*_.

CLC class name and code	Threshold (persons per km^2^)
Continuous urban fabric (111)	22110
Discontinuous urban fabric (112)	6431
Industrial or commercial units (121–142)	90
*Other artificial* (122–142)	31
Non-irrigated arable land (211)	31
Permanently irrigated land (212)	52
Rice fields (213)	10
Vineyards (221)	47
Fruit trees and berry plantations (222)	42
Olive groves (223)	56
Pastures (231)	40
Annual crops associated with permanent crops (241)	61
Complex cultivation patterns (242)	79
*Land principally occupied by agriculture* (243)	48
Agro-forestry areas (244)	7
Broad-leaved forest (311)	9
Coniferous forest (312)	6
Mixed forest (313)	8
Natural grasslands (321)	13
Moors and heathland (322)	13
Sclerophyllous vegetation (323)	7
Transitional woodland-shrub (324)	12
Sparsely vegetated areas (333)	19
*Uninhabitable natural areas* (331–332, 334–523)	0

As an additional limitation, only those land use classes in a given cell were used, which contained any man-made structures of particular kind. Three remote-sensing gridded datasets (100 m resolution) where used here; if no land use class in a cell possessed any structures from the first dataset, the second was used, then third if necessary, as follows:

1. Buildings;
2. Impervious surfaces;
3. Roads and streets.

Buildings and streets were obtained from European Settlement Map 2012 Release 2017 (https://land.copernicus.eu/pan-european/GHSL/european-settlement-map/esm-2012-release-2017-urban-green) and impervious surfaces from Imperviousness Density 2012. If no structures were present in the 1 km cell (as they were not detected in the satellite images), all land use classes were utilized.

The result of the calculation, however, is only the population per land use *L* in each 1 km grid cell *G*. Hence, the population had to be disaggregated further, and for that we used an approach similar to method M3. This method redistributes the population proportionally to the density of man-made structures. This variable has a range from 0%, which indicates completely natural surface, and 100%, which indicates land completely sealed by an artificial surface. The three datasets were used, primarily buildings from the European Settlement Map (ESM) 2012. If no buildings were indicated in a 1 km cell, imperviousness was used instead. In case no soil sealing was detected, roads and streets from ESM 2012 are used. This can happen mainly because ESM 2012 combined remote sensing data with multiple other sources (e.g. OpenStreetMap, European Union’s Urban Atlas and Tom Tom’s Tele Atlas), while Imperviousness Density 2012 is entirely a remote-sensing based product.

ESM and Imperviousness datasets have very high native resolutions (2.5 and 20 m, respectively). The version aggregated to 100-meter resolution was used for the disaggregation, while for computing the dependency between surface density and population was determined using data further resampled to a 1 km grid. In the process, average population density in grid cells with given artificial surface density could be calculated. The resulting dependencies can be approximated as power functions (Supplementary Fig. S2). Very few cells had very high average % of surface covered by structures, hence the functions were computed from values ranging from 1% to 16% (roads and streets), 64% (buildings) and 84% (impervious surfaces). Hence, the population *X*_*g*_ in 100-meter grid cell *g* is equal to:

6 $${X}_{g}=\frac{{Z}_{g}}{\sum {Z}_{g}}{Y}_{LG}{S}_{LG}$$

where *Z*_*g*_ is the population of grid cell *g* obtained from the power function divided by maximum population:

7 $${Z}_{g}=\frac{B{V}_{g}^{A}}{8000}$$

where *V*_*g*_ is the imperviousness in grid cell *g*. The maximum population was defined as 8000 as all three datasets reached peak population density around this value. The parameters *A* and *B* are indicated in Table 4.

**Table 4:** Parameters A and B in Eq. 7.

Dataset	*A*	*B*
Buildings	1.4399	19.4875
Impervious surfaces	1.5244	113.9358
Roads and streets	1.1658	38.8367

The population *X*_*g*_ is rounded, as population numbers need to be integers. Consequently, the population was added or subtracted by iteratively reducing population numbers in 100-meter cells starting with cells in which the smallest change in unrounded value would change the rounded value. In some cases, more than one 100-meter cell had equal values and the 1-km population couldn’t be matched. Then, population was added or subtracted by iteratively reducing population numbers by 1 at a time starting with 100-meter cells with the highest population. If again there were cases of multiple cells of equal values, 100-meter cells with higher % of area covered by structures were used. If no data was available or the % values were the same, the population is added or subtracted randomly within the equal cells.

**Fig. 2:**
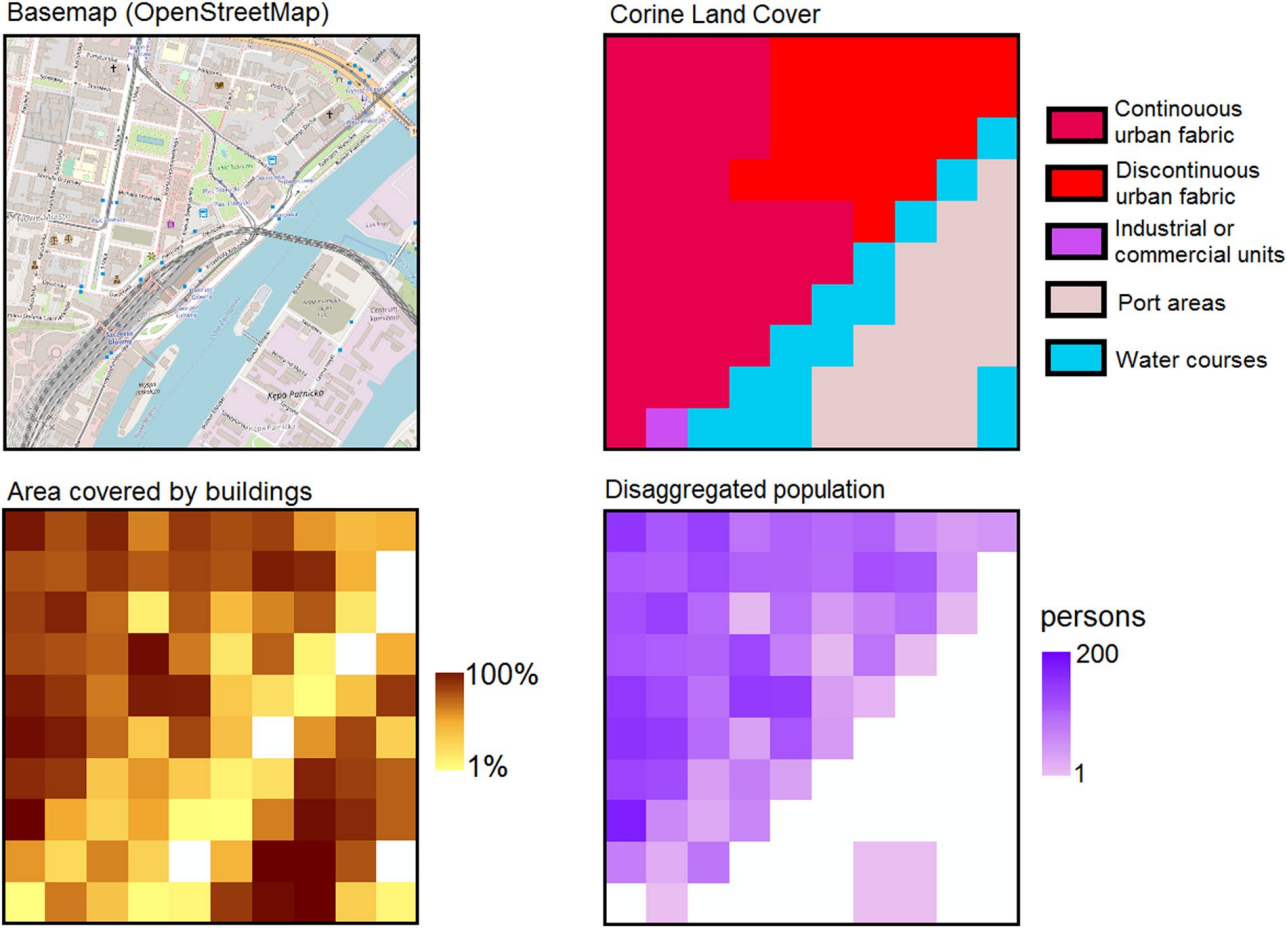
Disaggregation result and source data (population in the grid = 5230), contrasted with OpenStreetMap basemap for illustration only. Fragment of the city centre of Szczecin, Poland (NUTS region PL424). Basemap © OpenStreetMap contributors^40^. Distributed under the Open Data Commons Open Database License (ODbL) v1.0. Area covered by buildings from European Settlement Map 2012.

Example results of disaggregating the population for a single GEOSTAT grid cell is presented in Fig. 2.

#### Administrative boundaries

The HANZE v2.0 model redistributes population, land cover/use and economic variables separately for each subnational administrative unit (hereafter, “regions”). Administrative boundaries change frequently within countries over time. Rather than changing the definitions of regions at each timestep of the model, we adjust historical statistical data to a single benchmark classification. European Union’s classification of subnational units, the Nomenclature of Territorial Units for Statistics (NUTS), version 2010, is used in HANZE v2.0. The most detailed level of the classification, NUTS level 3 is applied. For this study we prepared a new, high-resolution vector dataset of NUTS regions due to the low precision and non-permissive licence of the semi-official NUTS map available through Eurostat (https://ec.europa.eu/eurostat/web/gisco/geodata/reference-data/administrative-units-statistical-units). We compiled the new dataset using only openly-available data from national geospatial agencies and OpenStreetMap^40^ (Table 5), with manual corrections of interregional boundaries made where necessary for alignment with NUTS 2010 classification. delineation of the coast in the original source datasets was adjusted to align it to the baseline CLC dataset. Further, as Kosovo and Bosnia and Herzegovina are not currently covered by NUTS, we artificially coded their administrative divisions in a manner consistent with the NUTS system. Finally, the vector dataset was converted into a 100 m raster grid. Our study’s domain contains 1422 regions in total. As noted, the input historical statistics that drive the model were recomputed where necessary to match our high-resolution map of NUTS regions.

**Table 5:** Sources of administrative boundaries.

Country	Provider
Austria	Bundesamt für Eich- und Vermessungswesen (https://www.data.gv.at/katalog/dataset/verwaltungsgrenzen-vgd-stichtagsdaten-grundstucksgenau)
Belgium	FPS Finance - General Administration of Patrimonial Documentation (https://www.geo.be/catalog/details/629ad470-71dc-11eb-af47-3448ed25ad7c?l=en)
Estonia	Estonian Land Board (https://geoportaal.maaamet.ee/eng/Spatial-Data/Administrative-and-Settlement-Division-p312.html)
Finland	The National Land Survey of Finland (https://www.avoindata.fi/data/en_GB/dataset/suomen-maakunnat-2021-vuoden-2018-maakuntakoodeilla)
Germany (except Mecklenburg-Vorpommern)	Bundesamt für Kartographie und Geodäsie (https://gdz.bkg.bund.de/index.php/default/nuts-gebiete-1-250-000-stand-31-12-nuts250-31-12.html)
Greece	Hellenic Mapping and Cadastral Organisation (http://geodata.gov.gr/en/dataset/oria-nomon-okkhe, http://geodata.gov.gr/en/dataset/oria-ota-pro-kapodistria)
Ireland	Ordnance Survey Ireland (https://data-osi.opendata.arcgis.com/datasets/51b0644d257143ba953f56b34558a4e0_0/, https://data-osi.opendata.arcgis.com/datasets/osi::local-electoral-areas-boundaries-ungeneralised-national-administrative-boundaries-2015/explore)
Italy (Sardegna only)	Istituto Nazionale di Statistica (https://hub.arcgis.com/datasets/inspire-esri::municipal-boundaries-of-italy-2019/about)
Norway	Kartverket (https://kartkatalog.geonorge.no/metadata/administrative-enheter-kommuner/041f1e6e-bdbc-4091-b48f-8a5990f3cc5b)
Poland	Główny Urząd Geodezji i Kartografii (https://www.geoportal.gov.pl/dane/panstwowy-rejestr-granic)
United Kingdom	Office for National Statistics (https://geoportal.statistics.gov.uk/datasets/c370f21b4b4649a5b6813bf48469836f_0/explore)
Other countries	OpenStreetMap contributors^40^

### Input socioeconomic data

The input database of historical socioeconomic statistical data was created by revising the data from HANZE v1.0. It contains data on the main socioeconomic drivers of exposure at regional level. The variables of the database are listed in Table 6. Further it contains fixed asset stock relative to GDP in six sectors, defined at country level. The database was compiled from 375 different sources (compared with 271 in HANZE v1.0): websites and publications of national statistical institutes and international agencies, working papers of national banks and economic research institutes, and academic research papers, dating from 1872 to present. Detailed information on the source of every single data point in the database, and transformations made to adjust data to NUTS version 2010 are described within the Excel datasets (see ‘Data Records’). The data was compiled every decade from 1870 to 1950, every 5 years until 2000 and annually until 2020. Compared to HANZE v1.0, the main changes are: improvement in the quality of data through inclusion of more data sources (Supplementary Fig. S3); addition of new countries (Albania, Bosnia and Herzegovina, Kosovo, Montenegro, North Macedonia and Serbia); addition of consumer durables (goods used by households for several years) as a category of fixed assets through integration of data and methods from Paprotny *et al*.^41,42^; addition of forest land cover data for the whole study area.

**Table 6:** List of input historical socioeconomic data used by the model.

Variable	Unit	Resolution
Population	Thousands of persons	NUTS 3
Urban fraction	Urban population as % of total population	NUTS 3
Persons per household	Mean number of persons	NUTS 3
Croplands	% of total area	NUTS 3
Pastures	% of total area	NUTS 3
Forests	% of total area	NUTS 3
Infrastructure	Area covered by road/railway sites in ha	NUTS 3
GDP	Million euro in constant 2020 prices	NUTS 3
GDP from agriculture	% of total GDP	NUTS 3
GDP from industry	% of total GDP	NUTS 3
Fixed assets in housing	% of total GDP	Country
Fixed assets in agriculture	% of GDP from agriculture	Country
Fixed assets in industry	% of GDP from industry	Country
Fixed assets in services	% of GDP from services	Country
Fixed assets in infrastructure	% of total GDP	Country

### Population and land-use model

The general approach, as noted in the introduction, is to modify the baseline population and land cover/use raster dataset for every timestep. This is done sequentially for different CLC classes and population groups (regional, urban, rural), so that a class that is modified in a given step doesn’t alter those that were modelled beforehand. The modelling steps are as follows:

1. Special cases (Dutch polders)
2. Sub-regional population redistribution
3. Urban fabric and urban population redistribution
4. Airports and reservoirs
5. Rural population redistribution
6. Industrial or commercial units
7. Road/railway sites
8. Construction sites
9. Other artificial land
10. Croplands and pastures
11. Burnt areas
12. Natural areas
13. Soil sealing degree adjustment

A summary of the modelling approach, and the rationale is explained, per land cover/use class from the CLC dataset, in Supplementary Table 1. It also highlights that artificial land use, though constitutes 5% of total land area, contains about 90% of population and fixed asset value, therefore the reconstruction of past exposure is largely limited to those areas. Detailed information is provided in the following subsections, as referenced in the numbered list above. It should be noted that the methodology is a refinement of methods largely used already in Paprotny *et al*.^32^.

#### Special case (Dutch polders)

The model includes one special case, due to its influence on exposure distribution in the Netherlands. The *Zuiderzeewerken* was a large-scale land-reclamation and flood-protection project, which resulted in the construction of large dikes and polders in the Zuiderzee between the 1920s and 1970s (Supplementary Fig. S4). Zuiderzee was closed in 1932 by a large dike, turned into a lake and further split in 1975 into IJsselmeer and Markermeer. Cities, infrastructure and farmland were created on the reclaimed land, mainly in the province of Flevoland. It has a population of more than 400,000 today, but before 1942 it consisted only of the small island town of Urk and the uninhabited island of Schokland (the province itself was only established in 1986). Therefore, all artificially-created land is removed from the land cover/use raster and turned into inland water (CLC 512) for years before the year of completion of individual Dutch polders. The population is also removed and not considered in the population and land-use redistribution for those years, hence this modelling step is done before all others.

#### Sub-regional population redistribution

Substantial redistribution of population within European countries occurred in modern times. Here, we model sub-regional (i.e. below NUTS3 level) population change for 1870–2020 based on empirical observations from a dataset of population change between 1961 and 2011 at the level of local administrative units (LAUs). We created the dataset for this study by merging tabular and spatial data produced in various years that is available through Eurostat^43^ and national statistical institutes (https://www.stat.gov.mk/OblastOpsto_en.aspx?id=2, https://www.stat.gov.rs/en-us/oblasti/stanovnistvo/, https://ec.europa.eu/eurostat/web/gisco/geodata/reference-data/administrative-units-statistical-units/communes). Details on how the data were created and their visualisation is provided in Supplementary Text S1. Population trends for around 109,000 LAUs indicate:

- Declining population in urban cores that are the most centrally located and densely populated parts of cities
- Rapid growth of suburban zones around urban cores
- Declining population of rural areas

The first two changes are largely driven by the change in number of persons per household. Even when the population of a city is stagnant, smaller families in each dwelling result in an increased demand for housing. Those extra dwellings had to be constructed mostly outside the urban cores, where the supply of housing is largely fixed. It has been shown^44–46^ that this trend has been present in major European cities since the early 19^th^ century, flattening the population density curve in relation to the distance from the city centres. At the same time, migration from rural to urban areas has reduced population in rural areas and exacerbated the growth of suburbs.

**Fig. 3:**
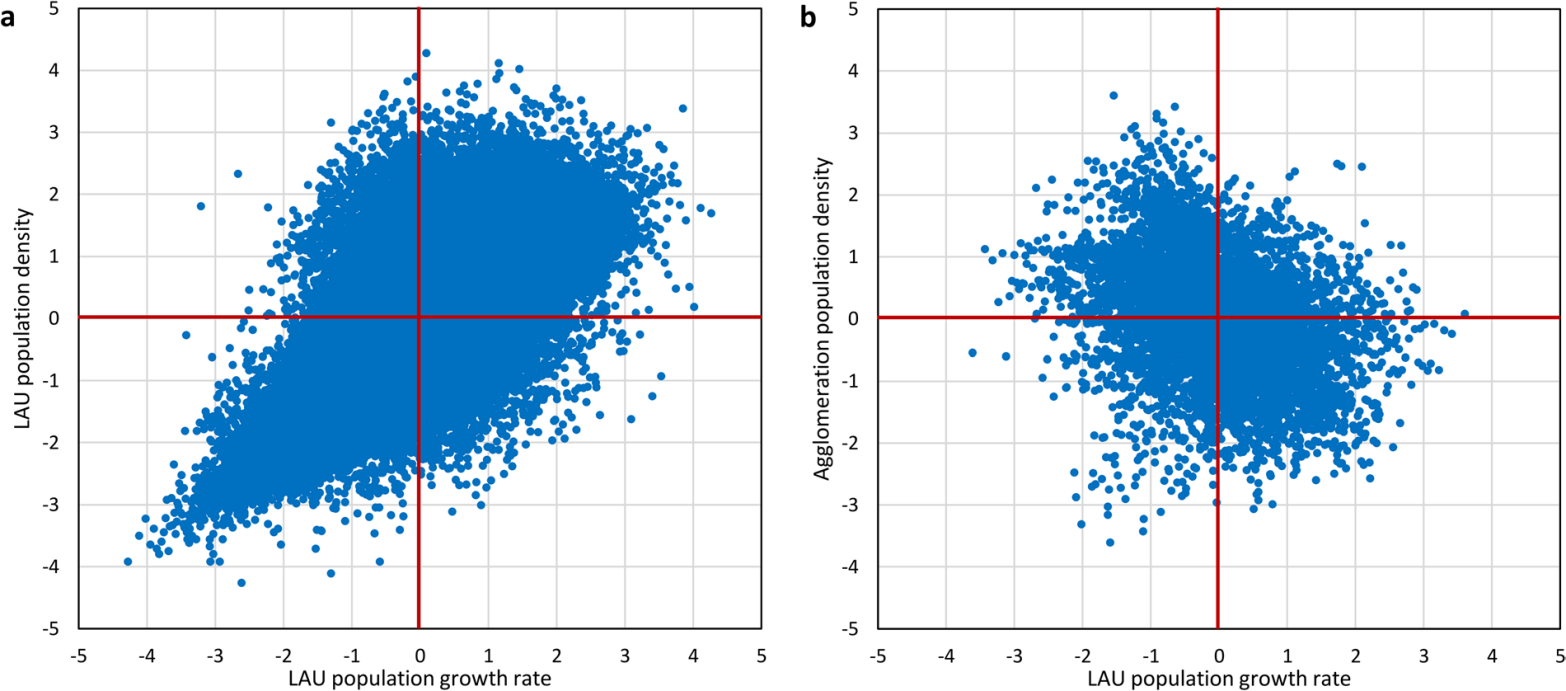
Empirical copulas of the dependency between population growth (1961–2011) relative to national growth, and population density (2011) in two different forms (a – local density, b – agglomeration density). Left copula (**a**) is applicable to population densities below and right (**b**) above 1500 persons per km^2^. Margins have been transformed to standard normal distributions.

Here, we model the rate of change of population within each NUTS3 region, where total population is defined by historical statistics, using the empirical relationship between population density and historical rates of change. To capture the uncertainty of the correlation, we use copulas that correlate population density from LAU data with population growth are applied (Fig. 3). A copula is, loosely, a joint distribution on the unit hypercube with uniform (0,1) margins. There are many types of copulas^47^, and we chose the optimal parametric copulas for this analysis by comparing different copulas using the “Blanket Test” based on Cramèr–von Mises statistic discussed by Genest *et al*.^48^. Due to the very different patterns of population change and high- and low-density LAUs, we use two copulas:

- a Gaussian copula using data from LAUs with population density below 1500 persons per km^2^ that correlates population density from LAU data with population growth (Spearman’s r = 0.69)
- a Frank copula using data from LAUs with a population density above 1500 persons per km^2^ that correlates “agglomeration density” with population growth (Spearman’s r = −0.36).

The “agglomeration density” per LAU is the average of the kernel density computed with the 1 km GEOSTAT population grid and a 10-km radius. Therefore, it indicates the size of the agglomeration that a high-density LAU is part of. This “agglomeration density” is a better predictor of population change than population density of individual LAUs. Only LAUs from NUTS3 regions with at least 10 LAUs were included to quantify the copulas to avoid using large, heterogenous LAUs. The threshold of 1500 persons per km^2^ for copula selection gave the best results in validation. Coincidentally, but unsurprisingly, it is also the threshold used to define high-density population clusters by Eurostat^49^, and in turn to classify LAUs as urban.

The LAUs from the Eurostat dataset applied here do not have complete coverage, have lower geometric accuracy than our NUTS3 vector dataset, and the size of LAUs varies substantially between countries. Therefore, a set of “virtual” LAUs (hereafter, “VLAUs”) was constructed. Every VLAU consists of an urban patch from Corine Land Cover 2012 and its nearest neighbourhood (Supplementary Fig. S5). Disaggregated population in 100 m resolution was then assigned to each VLAU to compute population density. For each timestep in the model, the copulas are sampled 10,000 times to obtain an estimate of annual population growth (geometric average of 50-year growth rate). The population of a VLAU in year *t* and 2011 is then:

8 $${P}_{t,VLAU}={P}_{2011,VLAU}{(1+A)}^{2011-t}$$

where *A* is the annual growth rate (in %) from the copula model. To avoid unrealistic changes, mainly for areas with very low population density, population growth is capped: −2.257% <*A*< 1.6464%, which corresponds to a 10-fold decline or 25-fold increase between 1870 and 2011. The analysis is done separately for each NUTS region, covering VLAUs or their parts located within a given region. After the population of all VLAUs was computed for a given NUTS region, the difference between the combined population of VLAUs and the population of the NUTS region in a given year as defined in the historical statistics is corrected by the same factor (relative to population) for every VLAU. The computation is done separately for urban and rural areas, i.e. those covered by urban fabric and all others, respectively. The share of urban population in each NUTS3 region is determined by the historical statistics.

#### Urban fabric and urban population redistribution

The population redistributed at sub-regional level is further adjusted spatially, separately for urban and rural areas. Assuming fixed supply of housing in already built-up areas, the population change in urban areas and expansion of those areas (i.e. urban fabric, or CLC classes 111 and 112) is driven by change in the total number of urban households. As the population has grown but the average number of persons per household has declined throughout Europe since the 19^th^ century, the demand for housing increased substantially. The movement of population to the edges of cities (suburbanization) is accompanied by the reduction of population density in the urban “cores” where a similar number of households contains a declining population stock. This process of flattening population distribution as a logarithmic function of distance from urban cores was quantified by Clark^44^ and many subsequent studies^45,46^.

By taking the total urban population *U* and average number of persons per household *H* (household size) from our historical statistics we can compute the total number of urban households *N*_*t*_ = *H*_*t*_/*U*_*t*_ in year *t* for every region. We simulate how the increase in *N*_*t*_ has caused urban fabric to expand through construction of new housing and related infrastructure in previously undeveloped areas. In rare cases, within recent years, there has been a decline in urban households over time. This so far has led to dwellings becoming vacant rather than a contraction of the area of the urban fabric.

The modelling operates by modifying, for a given timestep *t*, the population per urban fabric grid cell *P*_2011_ defined in the baseline population grid. This is done separately and independently for each VLAU, where the total baseline urban population is ${U}_{2011}=\sum {P}_{2011}$. The aim of this modelling step is to generate a new population grid, where $\sum {P}_{2011}$ matches *U*_*t*_, which in turn is the total urban population of a VLAU in timestep *t*. *U*_*t*_ is defined beforehand for each VLAU, as it is a proportional adjustment to the total population of all VLAUs in a given NUTS3 region, calculated already in section S2.2, to the total urban population of a NUTS3 region defined in the historical statistics. We therefore know the expected urban population in a VLAU and have to modify the population grid to reproduce the historical changes in the size of the urban population and change of their distribution within the cities. Changes in household size are taken from historical statistics at NUTS3 level. The procedure is done stepwise:

1. In every urban fabric grid cell in a VLAU, the grid-cell population *P* in year *t* is modified relative to the 2011 baseline to account for change in household size:
  9 $${P}_{t}={P}_{2011}\frac{{H}_{t}}{{H}_{2011}}$$
  where *H* is the average household size, determined for each NUTS3 region;
2. All grid cells in a region are ranked by distance from urban centres (explained further in the text), where the highest-ranked cells are the closest to any urban centre.
3. Surplus population *S*_*t*_ is calculated:

10 $${S}_{t}={U}_{2011}\frac{{H}_{t}}{{H}_{2011}}-{U}_{t}$$

where ${U}_{t}=\sum {P}_{t}$ is the total urban population in the VLAU. The modelling ends here if *S*_*t*_ = 0, but that is almost never the case. *S*_*t*_ is usually positive or negative, and indicates how many persons, after adjusting the population grid to the household size of historical level *H*_*t*_, have to be removed or added to the grid in order to match the historical total population *U*_*t*_. Depending on whether a year before or after the baseline is modelled, four combinations of *S*_*t*_ and *t* could be discerned, as indicated in Table 7.

**Table 7:** Possible combinations of surplus population St and timestep t, contrasted with illustrative examples taken from the database of historical statistics.

Case	Example (region, NUTS3 code and historical data)	Approach
**A** *S*_*t*_>0, *t*<2011	Potsdam, Germany (DE404) U_2011 = 152,656, H_2011 = 1.80, N_2011 = 84,668 U_1960 = 114,202, H_1960 = 2.96, N_1960 = 38,569 S_1960 = 136,500	The city more than doubled the number of households since 1960, which had to be accommodated through expanding the urban area with new housing districts. Hence, part of urban fabric in 2011 was created between 1960 and 2011 and has to be removed from the modelled exposure grid for 1960.
**B** *S*_*t*_<0, *t*>2011	Szczecin, Poland (PL424) U_2011 = 398,652, H_2011 = 2.40, N_2011 = 166,313 U_2020 = 389,660, H_2020 = 2.08, N_2020 = 187,247 S_2020 = −43,563	The city has increased the number of households since 2011, despite population decline. Hence, areas available for build-up in 2011 are converted into urban fabric in the 2020 grid to the extent needed to accommodate the new households.
**C** *S*_*t*_>0, *t*>2011	Vidzeme region, Latvia (LV008) U_2011 = 127,541, H_2011 = 2.53, N_2011 = 50,391 U_2020 = 111,053, H_2020 = 2.45, N_2020 = 45,309 S_2020 = 12456	Number of urban households in the region declined since 2011, which resulted in some dwellings being vacated. The urban fabric therefore doesn’t change in the 2020 grid, but the population is reduced in urban areas throughout the region.
**D** *S*_*t*_<0, *t*<2011	Liverpool, United Kingdom (UKD72) U_2011 = 466,415, H_2011 = 2.80, N_2011 = 203,701 U_1970 = 606,979, H_1970 = 2.26, N_1970 = 216,856 S_1970 = −36,819	Number of urban households in the city declined between 1970 and 2011, which resulted in some dwellings being vacated. The urban fabric therefore doesn’t change in the 1970 grid, but the population is higher in urban areas of the city in 1970.

These examples use data at NUTS3 level, but the calculation itself is done on the more detailed level of VLAUs.

In the two cases A and B, i.e. *S*_*t*_ > 0, *t* < 2011 and *S*_*t*_ < 0, *t* > 2011, the number of households, and therefore extent of urban areas, expanded over time. For timesteps before 2011 this means that some of the urban fabric has to be removed from the baseline land cover/use raster dataset (case A), while for timesteps after 2011 more urban fabric has to be added (case B). The changes in grid-cell population *P* will depend on the distance from urban centres *d*. The distance from urban centres used here is a weighted average of different measures of population centres (“combined distance”) in order to capture the multiple levels of hierarchy existing in urban networks. Five different datasets were tested and, based on a calibration process explained in the Supplementary Text S2 (the same as in Paprotny *et al*.^32^), four of those datasets were selected for the combined distance from urban centres. The datasets and their weights are as follows:

- Arbitrary centres of large agglomerations (more than 300,000 persons in 2018) and capital cities from United Nation’s 2018 Revision of World Urbanization Prospects (https://population.un.org/wup/), with a weight of 1.0
- Centroids of high-density population clusters^44^, with a weight of 1.5
- Centroids of cities included in the Urban Atlas 2018^44^, with a weight of 2.0
- Centroids of Corine Land Cover 2012 urban patches, with a weight of 0.5.

The combined distance is computed per each grid cell. Then, the modelling continues depending on the case:

- Case A: urban grid cells are iteratively removed going backwards from the base year 2011 starting with the lowest-ranked (*i* = 1), and their population is reduced by proportion *D*:
  11 $${P}_{t,i}={P}_{t,0}D$$
  Proportion *D* is based on the logarithm of distance from urban centres *d* in hectometres:
  12 $$D=\left(1-\frac{ln\left(d\right)}{ln\left(\arg \;{\rm{\max }}\left\{d\right\}\right)}\right)$$
  At each iteration the surplus is reduced by the amount of population redistributed:
  13 $${S}_{t,i}={S}_{t,i-1}-{P}_{t,0}(1-D)$$
  The calculation continues until *S*_*t,i*_ = 0. However, if at any iteration there is more population in grid cell(s) than remaining surplus, i.e.:
  14 $$\sum {P}_{t,0}(1-D)={S}_{t,i-1}$$
  the population is reduced by the available amount, split proportionally to grid cell population if there are more cells with the same rank:
  15 $${P}_{t,i}={P}_{t,0}\left(1-\frac{{S}_{t,i-1}}{\sum {P}_{t,0}}\right)$$
- Case B: cells where urban expansion most likely took place are identified using the land-use transition model described in step 10, starting with cells with the highest probability of transition. If more cells were given the same likelihood of transition to urban fabric than necessary to assign the additional population, the cells within that group were ranked according to distance from the urban centre. The population in the highest-ranked cells, i.e. iteration *i* = 1, is set to the maximum population per grid cell in the VLAU, reduced by proportion *D* from Eq. 12:
  16 $${P}_{t,i}=\arg \;{\rm{\max }}\left\{{P}_{t,0}\right\}D$$
  At each iteration the surplus is increased by the amount of population redistributed:
  17 $${S}_{t,i}={S}_{t,i-1}+{P}_{t,i}-{P}_{t,0}$$
  The calculation continues until *S*_*t,i*_ = 0. However, if at any iteration there is more population to be redistributed than the available surplus, i.e.:
  18 $$\sum {P}_{t,i}-{P}_{t,0} > -{S}_{t,i-1}$$
  the surplus is distributed equally between all cells that were modified until this iteration (denoted *n*):
  19 $${P}_{t,i}^{{\prime} }={P}_{t,i}\left(1-\frac{{S}_{t,n-1}}{\sum {P}_{t,i}}\right),i=\left\{1,\ldots ,n\right\}$$
  If there are no available empty grid cells in the VLAU, the population of all urban grid cells is increased proportionally in the same way as in Eq. 19.
- Case C and D: in those cases, the number of households declined over time, as some dwellings became vacant. The urban area remained unchanged, as urban fabric is not removed bar from very extreme cases. Before 2011, the population in all urban grid cells was added to the grid (case C), while after 2011, removed (case D). The population was increased/decreased proportionally to the population in a given grid cell in 2011 (as in Eq. 19).

Modelling the redistribution of population in urban areas is intertwined with change in urban fabric area. In cases A and B, the urban area changes as a result of the growth in urban household number, in contrast to cases C and D, where the urban fabric is kept unchanged. As urban fabric is closely related with high population density, urban fabric grid cells are only removed from (case A) or added to (case B) the baseline dataset if the changes to population density is large enough. Consequently, urban fabric is removed in timesteps before baseline year 2011 only if the population in a grid cell was reduced to less than 9 persons. For timesteps after 2011, only an increase of population to more than 81 per 100 m grid cell resulted in transition to an urban fabric class. Both thresholds were obtained by calibrating the model to match the magnitude of change observed in the CLC inventories (2000–2018). Between 2000 and 2012, urban fabric expanded by almost 1.88 million ha, while between 2012 and 2018 only by 98,676 ha, according to the CLC data. By setting the population thresholds through calibration, the model correctly represents the effect of urban population change on land-use type. As shown in the results, the calibration was effectively applicable back to the year 1900.

#### Airports and reservoirs

**Fig. 4:**
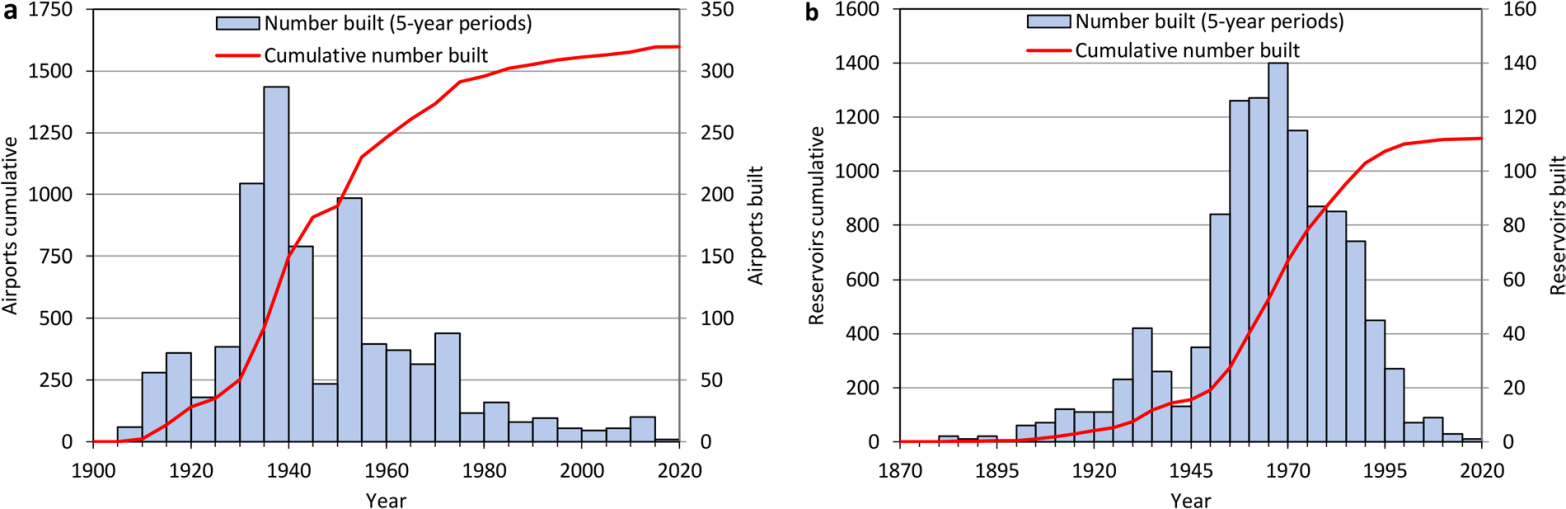
Number of (**a**) airports and (**b**) reservoirs built in the study area between 1870 and 2020 (5-year periods ending with the year indicated).

Airports and reservoirs are large elements of infrastructure that first appeared within this study’s timeframe. As the period of construction of those is usually well known and their number relatively small, they are removed or added to the baseline raster dataset based on the year of construction. We identified 1598 airports and 1121 large reservoirs (Fig. 4) in the study area by combining CLC datasets (CLC classes 124 and 512) with global databases of those objects (https://www.globaldamwatch.org/grand and https://ourairports.com/data/) and supplemented by web-based research of their history. Though HANZE v1.0 also included such data, due to the addition of new countries, the use of a revised CLC dataset and updates to the global airport and reservoir databases, we recompiled the data on airports and reservoirs from scratch. An airport or reservoirs that is removed from the baseline dataset enables other land-use types to fill the resulting empty space. An addition of such an object after 2011 removes any population that was present there in 2011.

#### Rural population redistribution

Rural population is recalculated separately for each VLAU, by adjusting the grid cell baseline population proportionally to its value, so that it equals the expected population in that area. For years before the baseline, areas from which urban fabric was removed are still considered urban for the purpose of this calculation. For years after the baseline, rural population in areas that transitioned to urban fabric is no longer considered rural, hence the adjustment is made only to remaining rural cells in the VLAU.

As the population is always recorded as integers, a discrepancy might arise from adjusting the population in grid cells and then rounding it. Therefore, the adjusted population in grid cells is first rounded using “floor” function, and then the grid cells with the highest remainders from dividing the unrounded values by 1 are rounded using “ceiling” function. The number of highest remainders is determined by the difference between the expected population in the VLAU and the total population of cells adjusted and rounded using “floor” function.

#### Industrial or commercial units

The area covered by large industrial/commercial facilities (CLC class 121) was assumed to change proportionately to GDP generated in a NUTS3 region by industry and services. Industrial grid cells located furthest from the centroids of industrial land use patches are removed first when going back in time. For timesteps after the baseline year, industrial grid cells closest to the centroids are added first. Industrial land use is only allowed to spread into uninhabited cells of some CLC classes: construction sites (133), agricultural (211–244) and certain natural areas (311–324 and 333). However, growth in GDP from industry and services is only partially driven by expansion of facilities, as the productivity of capital and labour tends to increase. Indeed, CLC 121 class has grown between 2000 and 2018 (based on CLC 2012 and CLC-Changes) by 16% in the study area, but GDP from industry and services increased by 32%. Therefore, the change in GDP from industry/services is scaled by an elasticity of 0.45, so that modelled changes between 2000–2018 in the study area have the same magnitude as observed in the CLC inventory. The industrial area *A* in region *r* and year *t* is as follows:

20 $${A}_{r,t}={A}_{r,2011}{\left(\frac{{G}_{r,t}}{{G}_{r,2011}}\right)}^{\varepsilon }$$

where *ε* is the elasticity and *G*_*r*_ is the regional GDP from industry/services according to the historical statistics at NUTS3 level.

#### Road and railway sites

The area covered by roads and railways (CLC class 122) before 2000 was assumed to change proportionately to the length of motorways and railways. Historical data on the length of this type of infrastructure was included in the input database. As infrastructure was built firstly in large urban and industrial zones, infrastructure grid cells (CLC class 122) located furthest from the urban centres are removed first when going back in time until the total area per region matches the value in the database. Conversely, grid cells closest to the urban centres are filled with infrastructure for timesteps after the baseline year. Infrastructure is allowed to spread only to particular CLC classes: construction sites (133), agricultural (211–244) and certain natural (311–324 and 333). However, construction sites were prioritised over other CLC classes; all ‘construction’ grid cells have to be used up before other CLC classes can be considered. The reason is that, apart from urban fabric or industrial sites already considered in previous steps, road and railway sites are the most frequent outcomes of construction activity. We found this pattern in the transitions of land-use in subsequent CLC inventories (2000–2018): almost half of the area of construction sites in the CLC inventory that transitioned to class other urban fabric or industrial sites (considered in previous steps) transitioned to infrastructure by the time of the next 6-year inventory.

#### Construction sites

Construction sites (CLC class 131) are by definition a temporary land use, typically for only a few years. The CLC inventory shows that 76–81% of construction sites transition to another land use during the 6-year periods between CLC datasets (2000–2006, 2006–2012, 2012–2018). Therefore, for years 2005–2011, their area was assumed constant, while for years 1870–2004 all construction sites were removed from the dataset. After 2011 they were allowed to transition into urban fabric, industrial sites, roads, railways, and airports (CLC 111–122 and 124), but otherwise kept unchanged.

#### Other artificial land

Green urban areas, sport and leisure facilities (CLC classes 141 and 142) are closely related to other artificial surfaces. Almost two-thirds of those CLC patches border either urban fabric, industrial sites, road/railway sites, or airports in the CLC 2012 inventory. Therefore, those patches of CLC classes 141 and 142 which bordered CLC classes 111–122 and 124 in the baseline dataset are removed if in a given timestep, if they do not border CLC classes 111–122 and 124 anymore due to application of previous modelling steps. Ports, mineral extraction, and dump sites (CLC classes 123, 131 and 132) are large elements of infrastructure like airports and reservoirs, but they are too numerous (almost 15,000 objects) and their history less traceable to apply the same approach as for airports. Therefore, they were kept constant at every time step and they did not interact with other land use classes, except in relation to polder (step 1) or reservoir (step 4) construction.

#### Agricultural land

The general concept of modelling changes of agricultural land was taken from HYDE^50^ dataset, i.e. local suitability for agriculture determines where this land-use class expands (most-suitable of available land first) and contracts (least-suitable falls into disuse first). Evolution in agricultural areas and increase in urban fabric after the baseline year was computed using a model utilizing a Bayesian Network (BN) that combines probability theory and graph theory in order to build and operate a joint distribution. The BN is trained with the CLC-Changes dataset, which records 1.2 million transitions involving patches of land larger than 5 ha, and CLC 2012 identifying land-use types that didn’t transition between 2000 and 2018. The CLC-Changes and CLC 2012 inventory were sampled to obtain 513,915 cases of transition and an equal number of land-use patches being stable between 2000 and 2018. For each location, information from different raster datasets were extracted as predictors of land-use changes: elevation and slope from EU-DEM^51^ dataset (https://ec.europa.eu/eurostat/web/gisco/geodata/reference-data/elevation/eu-dem/eu-dem-laea, agricultural suitability from Global Agro-Ecological Zoning (GAEZ) version 4 database (https://gaez-data-portal-hqfao.hub.arcgis.com/) and population dataset from steps 2 and 3. The sampling procedure and a list of all tested predictors is described in the Supplementary Text S3.

**Fig. 5:**
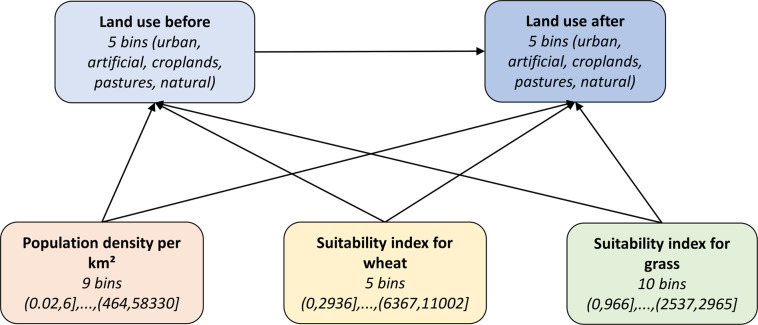
Bayesian Network for land-use transitions with 5 nodes and 7 arcs. The nodes indicate the number of bins of the discrete distributions and the intervals of the bins.

As the land use information is categorical, a discrete BN was used. Land-use classes were collected into 5 bins (urban fabric, other artificial, croplands, pastures, natural). The bin with natural land excludes non-utilizable land cover types (CLC 331–332, 334–335, 421–523), which are not allowed to interact with either artificial or agricultural land use. The BN model was constructed iteratively, starting with a simple three-node network, where the “old” land-use class is the parent of the “new” land-use class, and a single predictor variable is the parent of both land-use nodes. More complex BNs with more predictors were respectively validated against a disjunct subset of samples of transitions and non-transitions not used for training (see Supplementary Text S3 for sampling procedure and ‘Technical Validation’ for the final validation results). Iteratively, the best predictors, number of predictors and numbers of bins (into which continuous variables were discretized) were selected. Three predictors were chosen, all of which are parents of the two land-use nodes (Fig. 5):

- Population density per VLAU – 9 bins;
- Suitability index for wheat: output density (potential production divided by total grid cell area) for wheat under rainfed conditions and high input level – 5 bins;
- Suitability index for grass: agro-climatic potential yield for grass with an available water content of 200 mm/m (under irrigation conditions) and high input level – 10 bins.

The two agricultural suitability indices are from the GAEZ database, based on 1971–2000 climate. As the BN is quantified with a conditional probability table (CPT), this configuration results in the CPT having 11,250 cells. Therefore, no more variables were added to avoid too few data points quantifying cells of the CPT.

**Fig. 6:**
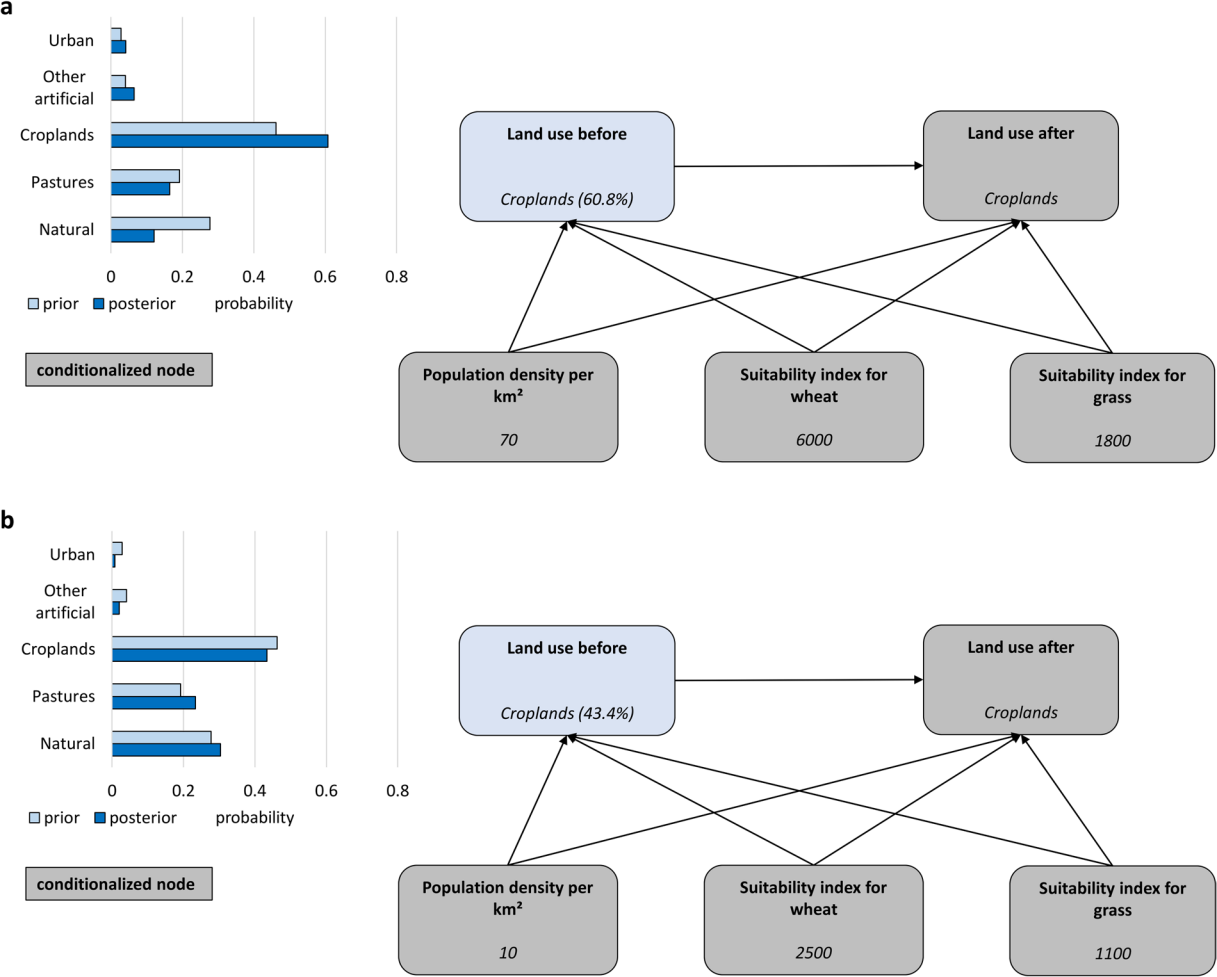
Example of a conditionalized Bayesian Network for land-use transitions. Panel (**a**) shows a highly-suitable area for croplands, and (**b**) an unsuitable one. The graph indicates the prior (situation in Fig. 5) and posterior (nodes in grey being conditionalized with values indicated) probability of previous land-use type (60.8% in (**a**) and 43.4% in (**b**) for being croplands).

Figure 6 shows an example of application of the Bayesian Network. In this case, we know the present-day land use (croplands) and that in some earlier timestep the total area of croplands in a NUTS3 region was lower than at present. Therefore, we want to know the probability that land-use was different from croplands across the grid cells located in the region. Figure 6a shows an area that was most likely a cropland before as well, due to relatively high population density and good suitability for agriculture. The area in Fig. 6b has lower suitability, which indicates a much higher probability that the area was used for other purposes than cropland. Consequently, the area in Fig. 6b will be ranked higher than area in Fig. 6a when selecting which grid cells of croplands will be removed from the raster dataset in order to match the total cropland area with historical statistical data.

The trained BN is used to generate probabilities of land-use transitions in nine cases, as follows:

- from non-urban to urban after the baseline year;
- from non-cropland to cropland after the baseline year;
- from non-pasture to pasture after the baseline year;
- from cropland to non-cropland after the baseline year;
- from pasture to non-pasture after the baseline year;
- from non-cropland to cropland before the baseline year;
- from non-pasture to pasture before the baseline year;
- from cropland to non-cropland before the baseline year;
- from pasture to non-pasture before the baseline year.

As noted in section 2.4.3, the BN handles the case of non-urban to urban transition after 2011. When the housing needs of the population result in expansion of cities, areas with the highest probability of transition from non-urban to urban land-use are build-up first. The BN is used in the same way for the remaining eight cases related to agriculture, i.e. they determine in which agricultural areas to add or remove so that the total area of croplands and pastures in the land cover/use raster dataset for a given time step matches the values obtained from historical statistics per NUTS3 region. This is done iteratively starting with patches of land with the highest probability of transition between given classes (e.g. non-pasture to pasture). Cropland redistribution is modelled first, then pasture is redistributed in the second step. Land still occupied by croplands after the first step cannot transition to pasture. However, land emptied by redistribution of croplands in the first step can transition to pasture in the second step.

We need to model transitions backward in time for timesteps before 2011. We partially remove urban fabric or roads/railways sites in case they occupied less land in the past and create an empty space, which croplands can occupy. Forward in time, it is a cropland to non-cropland transition. The probability of transition that is used to determine which cells to fill with croplands is the probability that a non-cropland cell was a cropland before. The same applies to pastures, with the condition that they cannot occupy cells already assigned to cropland. Transition of artificial surfaces still remaining at this step of the model to agricultural land-use is not allowed.

In the iterative land-use redistribution process, the number of grid cells with equal probability of transition might exceed the number of cells that need to be converted to match the total area in the historical statistics. This often happens as the predictors (GAEZ dataset and population density by VLAU) have relatively coarse resolution together with a small number of bins in which the data is divided. In order to derive exact 100-m grid cells from patches of land of equal probability, another predictor of agricultural suitability was added. Slope of the terrain is an important predictor, also used in the two agricultural suitability indices from FAO. It is available in the target (100 m) resolution as a continuous quantity from the EU-DEM elevation dataset. Lower slope indicates better suitability for agricultural activity, therefore 100 m cells of equal probability of transition are ranked according to the slope from lowest to highest. The appropriate number of highest-ranked cells is added/removed so that the total area of croplands or pastures exactly matches the total area in the historical statistics.

#### Natural areas

Natural areas contain less than 1% of population and fixed assets, therefore limited modelling is done for those classes. Areas where vegetation has burned down (typically forests) are by definition a temporary land use. Burnt areas are very short-lived: almost none of the land cover patches in this class (CLC 334) in 2012 were present in either 2006 or 2018 CLC inventories. For years 2007–2017, burnt area was assumed constant, while for years 1870–2006 and 2018–2020 all burnt areas were removed from the dataset. As almost all burnt areas are formerly or subsequently CLC classes 311–324, this modelling step is done after redistributing agricultural areas. Still, there is no exposure in burnt areas, and very little exposure in preceding land-use, except for rare cases.

Natural areas are what remains after modelling artificial, agricultural and burnt areas as well as reservoirs and special cases. Natural areas would cover the entire continent without human activity. Therefore, if land becomes unoccupied as a result of the modelling, it is assigned the same natural land cover that is typical in its nearest neighbourhood. Typical natural vegetated land cover (classes CLC 311–324 and 411–422) is defined as the most frequently occurring one within the VLAU. The calculation is done separately for forests (CLC 311–313) and other natural land (CLC 321–324 and 411–422), and the more frequent of the two groups is used. If there is no natural cover in the VLAU, the dominant vegetated land cover of the applicable NUTS3 region is used. If no vegetated land cover was located in the NUTS3 region, the unoccupied land was assumed to be covered by transitional woodland-shrub (CLC 324), as it is the most common non-forest natural land in the study area.

After the first allocation, the total area of forests is compared with the historical data in the NUTS3 database. If there is too much forest area in a given NUTS3 region, the land that was allocated to forest in this step is iteratively converted into the most frequent non-forest class, starting with the most-densely populated VLAU. Conversely, if there is not enough forest land, cells that were allocated to non-forest vegetation in this step are iteratively converted into the most frequent forest class, starting with the least-densely populated VLAU.

All other natural land, without vegetation and usually prohibitive to construction or agriculture, i.e. beaches, dunes, sands (CLC 331), bare rocks (CLC 332), glaciers and perpetual snow (CLC 335), intertidal flats (CLC 423), and water (CLC 511–523), were kept constant throughout. Patches of those types of land cover are removed from the dataset only in connection to reservoir (step 4) or polder construction (step 1).

#### Soil sealing degree adjustment

Changes in soil sealing are entirely based on land-use transitions, therefore this step is carried out after land-use modelling, but before economic data disaggregation. Soil sealing in the baseline raster dataset is increased to the average value for a given CLC class (Table 8) when non-artificial land transitions to artificial, unless it is already higher than that value. For the backward in time calculation for timesteps before 2011, wherever land that is currently artificial is changed to agricultural, the degree of soil sealing is reduced alongside to 1%. Similarly, it is reduced to 0% in cases when agricultural or artificial land is changed to natural land.

**Table 8:** Average soil sealing in the Imperviousness 2012 dataset per selected types of Corine Land Cover classes.

CLC classes	Average soil sealing in 2012
Urban fabric (111–112)	28%
Industrial or commercial units (121)	45%
Road and rail networks (122)	29%
Airports (124)	20%
Agricultural areas (211–244)	1%
Natural land (311–523)	0%

### Economic data disaggregation

The disaggregation of economic data follows dasymetric mapping methods, similar to applied in European^52^ or global^53^ studies, including HANZE v1.0. Several revisions to the latter were introduced (Table 9). Regional GDP is split partially proportionally to population and partially according to land-use (with soil sealing where appropriate). In this way, both labour (part of the total population) and capital (connected to land-use) input to GDP is represented. Labour share of GDP in advanced countries is about 60% and has been relatively stable over time^54^. Hence, 60% of GDP is disaggregated according to population and the remaining 40% using land use. Fixed assets in absolute terms per region are computed by multiplying regional GDP, or a sector thereof, by the respective wealth-to-GDP ratio for each sector, as defined by variables “Fixed assets” (Table 6). Housing and consumer durables are distributed according to total population, as they are most closely related to population distribution. Other assets, related to economic activities, are distributed to appropriate land use classes, proportionally to the degree of soil sealing. Finally, infrastructure is distributed to urban and industrial land (CLC classes 111–121) proportionally to the area covered by roads and streets, and to roads/railways, ports, and airports (CLC classes 122–124) proportionally to the degree of soil sealing.

**Table 9:** Disaggregation of economic variables by population, land use classes (CLC = Corine Land Cover class codes) or soil sealing degree.

Variable	Category	Population	Land use	Soil sealing
GDP	Agriculture excl. forestry	Population in CLC 211–244 (60%)	CLC 211–244 (40%)	—
	Forestry	Population in CLC 311–313 (60%)	CLC 311–313 (40%)	—
	Industry excl. mining	Total population (60%)	CLC 121 (40%)	yes
	Mining	Total population (60%)	CLC 131 (40%)	—
	Services	Total population (60%)	CLC 111–124/133/141/142 (40%)	yes
Wealth	Housing	Total population	—	—
	Consumer durables	Total population	—	—
	Agriculture excl. forestry	—	CLC 211–244	yes
	Forestry	—	CLC 311–313	yes
	Industry excl. mining	—	CLC 121	yes
	Mining	—	CLC 131	yes
	Services	—	CLC 111–121/133/141/142	yes
	Infrastructure	—	CLC 111–124	yes (streets and roads for CLC 111–121)

There are also additional assumptions on disaggregation of GDP and wealth for two sectors. Agricultural sector encompasses farming, fishing and forestry, while the industrial sector includes mining, manufacturing, and utilities. A detailed breakdown of those subsectors is not available at regional level for GDP, or at all for fixed assets, except for a small number of countries. Hence the regional GDP and wealth from forestry and mining was estimated by computing “efficiency indices” at national level. The forestry index was compiled by computing GDP from agriculture (without forestry) at national level per ha of agricultural land from CLC and GDP from forestry per ha of forest land. Those values were computed for the year 2000 for all countries and presented as efficiency of the forest economy relative to other agriculture in %. This ratio was used to compute the relative share of forestry in regional GDP in any given year based on land cover/use modelled for that year:

21 $${G}_{f,r,t}={G}_{af,r,t}\frac{{A}_{f,r,t}{E}_{f,c}}{\left({A}_{a,r,t}+{A}_{f,r,t}{E}_{f,c}\right)}$$

where *G* is GDP, *A* is area covered by land cover/use in a particular sector, *E*_*c*_ is the efficiency index for country *c*. The forest sector is denoted by *f*, agricultural sector (without forestry) by *a*, NUTS3 region by *r* and timestep by *t*. Agricultural GDP without forestry is therefore:

22 $${G}_{a,r,t}={G}_{af,r,t}-{G}_{f,r,t}$$

The wealth-to-GDP ratio for agriculture is used for both forestry and other agriculture. Mining and quarrying are split from the remaining industrial activities (manufacturing and utilities) using a mining efficiency index, calculated like the forest index. In the same way, it uses the proportion of mining areas (CLC 131) relative to industrial areas (CLC 121) in each NUTS3 region and timestep to disaggregate the two sectors. Equations 21 and 22 are applicable with substituting the different sectors and land-use types. The wealth-to-GDP ratio for industry is used for both mining and other industries.

## Data Records

The dataset available on Zenodo^55^ consists of three components (Table 10). First is a set of GeoTIFF rasters covering the whole domain of 42 countries. Each raster has a resolution of 100 m and the standard European spatial reference ETRS89/LAEA (EPSG:3035). There is a total of 195 raster datasets, one each of the five variables (land cover/use, population, GDP, fixed assets and soil sealing) and 39 timesteps (decennial 1870–1950, 5-yearly 1950–2000 and annual 2000–2020). Economic variables are valued in euro (EUR) using constant price level and exchange rates from other currencies in year 2020. For quicker visualization of the land cover/use grids, legend files for ArcGIS and QGIS are also included in the repository.

**Table 10:** List of output datasets of HANZE v2.0 model stored in the repository (10.5281/zenodo.7885990)^55^.

File	Format	Variables/contents
**Raster datasets (YYYY** = **year) for all NUTS 3 regions, separately for each timestep (decennial 1870**–**1950, 5-yearly 1950**–**2000 and annual 2000**–**2020)**		
CLC_YYYY	8-bit GeoTIFF	Land cover/use type, 44 classes according to Corine Land Cover classification
Pop_YYYY	16-bit GeoTIFF	Total population per grid cell (in persons)
GDP_YYYY	32-bit GeoTIFF	Gross domestic product (GDP) per grid cell per year (euro in constant 2020 prices)
FA_YYYY	32-bit GeoTIFF	Wealth (fixed asset value) per grid cell (euro in constant 2020 prices)
Imp_YYYY	8-bit GeoTIFF	Soil sealing degree (%)
**Uncertainty estimates of exposure for all timesteps in NUTS3 regions, separate set of files (“TYPE”) for riverine and coastal flood hazard (100-year return period)**		
Exposure_TYPE_hazard_zone	CSV file	Formatted data with the following columns: NUTS: region code Variable: population, GDP or fixed assets Percentile: 5^th^, 20^th^, 50^th^, 80^th^ or 95^th^ 1870…2020: year
**Historical statistics database**		
NUTS3_database_population_land_use	Excel file	Input land use/cover and population data (NUTS 3 or country level), see Table 11
NUTS3_database_economy	Excel file	Input and auxiliary economic data (NUTS 3 or country level), see Table 12

The second part are uncertainty estimates of past exposure to floods. It was created using the model’s ability to compute probabilistic outputs based on uncertainty of reconstructing past population distribution and land-use transitions (modelling steps 2 and 10). However, the uncertainty distributions of individual grids cells are not independent, but highly correlated, therefore it was not possible to present the uncertainty bounds in the same format as the “best estimate” raster datasets. Consequently, they have to be computed by sampling the model and aggregating each iteration for defined hazard zones. The uncertainty estimates in the repository were created using flood hazard maps for 100-year return period events, taken from Paprotny *et al*.^56^ for coastal hazard and Alfieri *et al*.^57^ for riverine hazard. The 5^th^, 20^th^, 50^th^, 80^th^, 95^th^ percentile of population, GDP and fixed asset value for all 39 timesteps is contained in separate files for each NUTS 3 region, variable and type of hazard.

The input data of the HANZE v2.0 model that were used to generated this dataset are listed in Supplementary Tables S3–S6. Of particular interest for researchers are the input databases of historical land use, population, GDP, fixed assets as well as other demographic and economic variables. The statistics are mostly at NUTS 3 level (some variables at country level), compiled in this and previous (HANZE v1.0) study^32^ by harmonizing almost 400 separate sources of data, therefore it is also included as the third part of the output data (Tables 11, 12).

**Table 11:** Contents of the historical statistics database for population and land use (10.5281/zenodo.7885990)^55^.

Table	Variable/unit	Table structure
Population	Thousands of persons	Code – NUTS3 region code Name – NUTS3 region name 1870…2020 – data by year
Urban fraction	Urban population as % of total population	As above
Persons per household	Mean number of persons	As above
Croplands	% of total area	As above
Pastures	% of total area	As above
Forests	% of total area	As above
Infrastructure	Area covered by road and rail infrastructure in ha	As above
Census information	Additional information on the 2011 censuses, which are the baseline population figures	Code – NUTS0 country code Name – country name Date – census date Type – census type Source – method of collecting population data GEOSTAT accuracy – information on gridded data production methods
Airports	Airports identified in the CLC data	CLC – Corine Land Cover 2012/2018 vector polygon code Name – airport name Year – year of construction NUTS3 – NUTS3 region code ICAO – airport ICAO code IATA – airport IATA code
Reservoirs	Reservoirs identified in the CLC data	CLC – Corine Land Cover 2012/2018 vector polygon code Name – name of dam Year – year of construction GRAND – reservoir code in GRanD database
Sources	Information on sources and methods used to collect the data	Country – country name Variable – variable (per country) Sources – details on sources for all years (per variable and country)
References	List of all works cited in “Sources”	Source – bibliographic reference

**Table 12:** Contents of the historical statistics database for economic data **(**10.5281/zenodo.7885990)^55^.

GDP	Million euro in constant 2020 prices	Code – NUTS3 region code Name – NUTS3 region name 1870…2020 – data by year
GDP from agriculture	% of total GDP	
GDP from industry	% of total GDP	
Fixed assets	% of total GDP	Code – NUTS0 country code Name – country name Category – type of fixed asset and unit of measure 1870…2020 – data by year
	Housing (% of GDP)	
	Consumer durables (% of GDP)	
	Agriculture (% of GDP from agriculture)	
	Industry (% of GDP from industry)	
	Services (% of GDP from services)	
Sector indices	Forest economy efficiency relative to agriculture (%)	Code – NUTS0 country code Name – country name Forest, mining – sector indices for year 2000
	Mining economy efficiency relative to industry (%)	
GDP deflator	Index, base year = 100	Code – NUTS0 country code Name – country name 1870…2020 – data by year Unit – unit of measure (2020 = 100, 1990 = 100 or 1913 = 100)
Currencies	List of all currencies used in the period	Code - NUTS0 country code Name - country name Currency - currency name Code1 - three-letter currency code Code2 - ISO 4217 numeric code Start date - date when currency first entered circulation End date - data when currency was withdrawn from circulation Conversion – conversion factor between new and old currency Note – important annotations about the currency
Currency conversion	Conversion factors of all currencies to 2020 euros (euro = 1).	Country – NUTS0 country code Currency – currency code Code – merged NUTS0 and currency code Conversion – conversion factor
Sources	Information on sources and methods used to collect the data	Country – country name Variable – variable (per country) Sources – details on sources for all years (per variable and country)
References	List of all works cited in “Sources”	Source – bibliographic reference

## Technical Validation

Validating high-resolution exposure data is a major challenge due to limited availability of comparable observational datasets^29^. Here, we utilise available population and land-use data for validation and further compare the results with other published modelled datasets. Validating the disaggregation of economic data is currently not possible due to the complete lack of observational data.

### Population disaggregation

There is a general lack of very high-resolution population reference data, partially due confidentiality reasons^29^. The GEOSTAT 1 km grid – our input for population disaggregation – is already artificially distorted in some grid cells due to “confidentiality treatment”. It provides the highest resolution available for an observational product. Therefore, we prepared an alternative disaggregation of 1 km population to 100 m using floor space of residential buildings as predictor, rather than aggregated land-use and soil sealing data. We use high-resolution building vector data (https://www.geoportal.gov.pl/dane/baza-danych-obiektow-topograficznych-bdot) for municipalities threatened by sea level rise in Poland previously applied by Paprotny and Terefenko^58^, as the data they used were accurate as of 2012/13, which is close to our baseline year. Within each 1 km grid cell completely within the validation area, we computed the residential floor space in m² using the area of residential buildings, multiplied by the number of stories, per 100 m grid cell of our high-resolution population grid. The population was distributed proportionally to floor space in each 100 m cell. For the calculation we excluded collective-living facilities in which people do not normally register addresses, summer houses or abandoned buildings.

We compare our modelled results with the alternative disaggregation and a previously-published 100 m disaggregation of GEOSTAT called GHS^59^. Those grids were then intersected with pan-European flood hazard maps for coasts^56^ and rivers^57^. We found that both the HANZE and GHS grids smooth the spatial distribution of population too much, as indicated by false positive ratios (Table 13). Almost 40% of populated cells in HANZE have no population indicated in the benchmark dataset, though in half of those cases the indicated population is only one or two persons. The false positive ratio is higher in GHS than in HANZE and above 40%. Conversely, HANZE rarely indicates no population wrongly: only 3.5% of cells not populated in HANZE are populated in the benchmark dataset. This is less than the 4.7% in GHS (false negative ratio in Table 13). Exposure within river and coastal flood zones for municipalities of the Polish coastal zone (with at least 30 persons exposed) was mostly represented well, with a median error of above 10% in HANZE. HANZE achieved better results than GHS for river flood hazard zones, though exposure to coastal flood was better modelled by GHS. A final check of the datasets was carried out by binning the population per 100 m grid cells in intervals of increasing by factor of 2: [0,1], [1,2], [2,4], [4,8], [8,16] etc. We found that the population per cell in HANZE was within +/− 1 interval of the validation dataset in 53% of the cases, which is better than 44% computed for the GHS dataset.

**Table 13:** Accuracy of population disaggregation to 100 m resolution in this study and in the GHS grid, compared with the benchmark dataset (alternative disaggregation using residential building vector data).

Metric	This study	GHS
False negative ratio	3.5%	4.7%
False positive ratio	37.5%	40.9%
Median absolute error in exposed population by municipality for coastal floods	11.6%	8.3%
Median absolute error in exposed population by municipality for river floods	10.3%	12.1%

### Population change

The most detailed level at which validation of the modelled population changes is possible is the municipality level. We obtained two reference datasets for this purpose. First, we use the pan-European dataset (1960–2010) with population data by local administrative unit used to quantify our model (see Supplementary Text S1). Secondly, we assembled a dataset with a longer timespan for Austria based om historical census data recomputed to present-day municipalities by Statistik Austria (https://www.statistik.at/datenbanken/statcube-statistische-datenbank) and combined with a vector dataset of their boundaries (https://www.data.gv.at/katalog/dataset/verwaltungsgrenzen-vgd-stichtagsdaten-grundstucksgenau). The resulting reference population dataset covers the entire time span of this study (1870–2020) and 2117 units: all municipalities plus the districts of Vienna (Supplementary Fig. S9). For further comparison we use the HYDE 3.2 dataset^25^, recomputed from 5′ resolution to municipalities. Both HANZE and HYDE utilise subnational population data that is disaggregated both in space and time, making them the closest comparable exposure products.

**Fig. 7:**
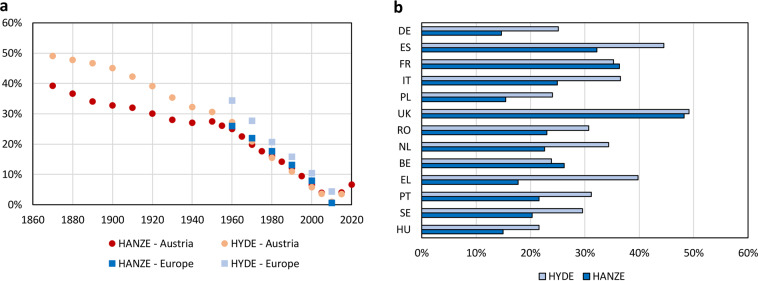
Accuracy of population change estimation compared with HYDE dataset. The error metric (in %) indicates average absolute difference in modelled and observed population per LAU relative to observed population in a given year (**a**) and across countries in 1960 (**b**).

Accuracy of population change at the level of local administrative units (LAUs) was analysed using the average absolute difference in modelled and observed population per LAU relative to observed population in a given year. As Fig. 7a indicates, error grows as more time elapses from the baseline year, reaching an average of about 20% by 1960 (in both validation areas) and 40% by 1870 (in Austria). However, the majority of LAUs are small rural communities, with more than half of LAUs in Europe having a population of less than 1000 in 1960, and a third in Austria in 1870 (Supplementary Table S7). Both European and Austrian LAUs have changed population by more than a factor of two since 1960 and 1870, respectively. Therefore, absolute errors are mostly small (less than 200 persons in half of the LAUs in Europe). In larger LAUs, the relative errors are smaller, though in Austria in 1870 errors in particular the districts of Vienna dominated the largest grouping of LAUs. The error varies by country (Fig. 7b) and is partially connected to the size of LAUs (relatively small in France or the United Kingdom, large in Poland and Greece) or the number of LAUs per NUTS3 region (on average 381 in France, but only 28 in Germany). Countries with large LAUs or small NUTS3 regions show less significant errors. In general, HANZE shows lower errors than HYDE, with small exceptions, for instance in Austria after 1980, though the population changes in that period were rather small compared to previous decades. From all major countries, France and Belgium show higher errors in HANZE than in HYDE in estimating population in 1960, while among small countries this only occurs for Luxembourg and Slovenia.

### Land cover/use change

Validation of land cover/use change is based on samples of CLC and CLC-Changes from 2000 to 2018. We use such sampled to quantify the land-use transition model in step 10, but for validation we have drawn an additional, non-overlapping random sample of transitions (CLC-Changes) and non-transitions (the same class in different CLC inventories). A total of 97,790 samples each for transitions and non-transitions were used (see Supplementary Text S3 for details of the sampling procedure). The net amount of land that transitioned is known from historical statistics, hence a defined number of cells with the highest probability of transitioning according to the Bayesian Network model is selected. A validation metric can therefore be the percent of top-ranked cells, up to the amount that is known to have transitioned between defined land-use classes, that was correctly identified by the model. As the number of cells in different land-use classes varies, the success rate has to be contrasted with a random result, i.e. the success rate of randomly picking land-use cells as transitioning. The results are presented in Table 14. In all considered cases of land-use transitions, the model’s success rate in correctly identifying transitioning cells in the validation dataset is much higher than if cells were picked randomly.

**Table 14:** Correctly identified (success rate) transitions of land use in the validation dataset.

Transition	Modelled	Random result
Other to urban	32%	7%
Other to cropland	51%	20%
Other to pasture	23%	5%
Cropland to other	62%	33%
Pasture to other	36%	13%

Overall land cover/use modelling results in HANZE are compared with HILDA^22–24^. It has a resolution of 1 km, containing changes in land cover/use for six classes (aggregated from CLC classification) from 1900 to 2010 over a domain of 29 countries and territories. HILDA is primarily a model that reallocates land-use based on aggregate historical statistics and probability maps, similarly to both HANZE and HYDE. However, it also integrates, where possible, digitised historical maps. On the other hand, HILDA is primarily focused on agricultural land and its interaction with natural vegetation, which is of less interest in this study due to relatively low exposure related to those land cover categories.

**Fig. 8:**
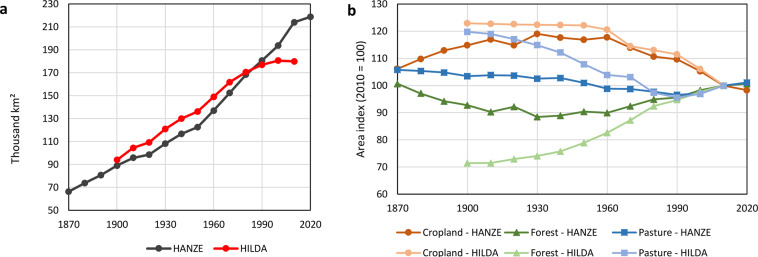
Artificial surfaces in thousand km^2^ (**a**) and other main land use classes relative to year 2010 (**b**) in HANZE (this study) and HILDA, for the 29 countries and six aggregated CLC land cover/use classes available in HILDA.

HILDA indicates some important similarities with HANZE. The area of artificial surfaces has a very similar trend in the two datasets between 1900 and 1990 (Fig. 8a), even though HANZE was calibrated only for years 2000–2018. This indicates that the underlying processes do not strongly change over time and the model is also applicable to times before the calibration period. HILDA indicates almost no growth in artificial surface area after 1990, in contrast to HANZE. However, CLC and other datasets indicate strong growth. For instance, the LUCAS land-use survey data (https://ec.europa.eu/eurostat/statistics-explained/index.php?title=LUCAS_-_Land_use_and_land_cover_survey) for 23 countries show that artificial surface expansion of 11% in only nine years (2009–2018). Cropland change is similar in both datasets (Fig. 8b) as largely similar data sources were used after 1950. Before that date HANZE used various national statistical data, while HILDA interpolated historical statistics or maps from 1950 backwards to 1900. The datasets differ significantly for pastures and forests. HILDA indicates a strong decrease in the area covered by pastures, which are replaced mostly by forests. By contrast, the historical statistics collected for HANZE do not indicate a similar pattern found in HILDA. However, it could be also partially because there is no detailed model for transitions between forest land cover and other natural land in HANZE. Therefore, reforestation of various natural land types that fall under “pastures” category in HILDA is not captured by our model. Due to the very low exposure and negligible change in that exposure due to such transitions, we do not address them with a more detailed model. Finally, HILDA surprisingly indicates a decline in area covered by water, which is opposite to HANZE, where reservoir construction leads to the overall expansion of water bodies in Europe.

### Soil sealing degree change

**Fig. 9:**
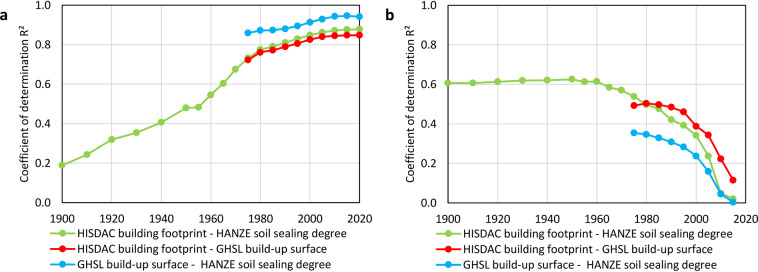
Comparison of correlation between three datasets of artificial land cover (% of total area) aggregated to municipalities of Spain, (**a**) at given timestep or (**b**) differences between a given timestep and 2020.

No dataset covers soil sealing from any source over a longer time period. Some comparison could be made using a recently published dataset of building footprints in Spain (HISDAC^60^), covering years 1900–2020. The dataset is based on cadastral data that record the year of construction of buildings. Even though buildings form only a part of sealed surfaces, we compared average coverage of buildings and sealed surfaces for 8109 municipalities in European Spain (i.e. without Ceuta, Melilla and the Canary Islands) between 1900 and 2020 from HANZE and HISDAC. As additional comparison, we computed average build-up surface between 1975 and 2020 from the Global Human Settlement Layer (GHSL^61^), which derives this information from satellite imagery. In this domain, average coverage in 2010 (closest to the baseline year) is 1.17% for HANZE (soil sealing), 0.64% for GHSL (build-up surfaces) and 0.50% for HISDAC (building footprint). Despite the difference in definition, HANZE is more correlated with both datasets than HISDAC with GHSL (Fig. 9a), which should be more closely related. Moving further into the past, the correlations decrease, but for 1975–2020 the correlation between HISDAC and HANZE remains higher than between HISDAC and GHSL. For short-term changes (Fig. 9b), all three datasets show very low correlation, with increases with the time period over which the changes are analysed. By 1975, the changes in HISDAC are more correlated with HANZE than with GHSL. The trends in building footprints in HISDAC show stable correlation with trends in HANZE soil sealing for the period 1900–1960. Considering that HANZE explicitly doesn’t model (partially or wholly) changes in artificial surfaces with relatively low exposure (e.g. minor roads and railways, dump sites, urban recreational spaces, etc.), the results indicate that the model can capture long-term, intra-country variation of soil sealing.

## Usage Notes

The primary goal in creating HANZE dataset of exposure was computing exposure to past natural disasters and then adjusting the reported losses for various events to a common benchmark year (commonly known as “normalization” of loss data^17^). Paprotny *et al*.^16^ used HANZE to normalize losses for 1564 flood events between 1870 and 2016. In this new iteration of HANZE, the code is publicly available for further analyses. All input datasets (Supplementary Tables S3–S6) are available in a repository^62^, hence the user only needs only to download them and change the defined path to the folder with data. Then, the code^63^ can be run using the basic options embedded in the code, which are:

- generating five exposure rasters (land cover/use, population, GDP, fixed assets, soil sealing) in GeoTIFF format and 100 m resolution. A single year or multiple years out of those included in the database (10-yearly 1870–1950, 5-yearly 1950–2000, annually 2000–2020) could be run. Also, all NUTS3 regions could be included, or only a single NUTS3 region, or several regions. The output exposure datasets are also available in the repository, as even if the model is rather efficient given its resolution (about one hour for one timestep for all NUTS3 regions), computing all 39 timesteps of the study would require large resources or time.
- computing exposure (population, GDP, fixed assets) per hazard zone. A raster file with the same spatial extent as the other input raster files is needed for this. Example files are provided in the repository, which enable reproducing the analysis presented in this section. Using this option, a text file with data (for years defined by the user) is saved separately for each NUTS3 region.
- computing exposure with uncertainty bounds per hazard zone. This is an extension of the previous option, which saves a text file per region and variable (population, GDP, fixed assets) with the 5^th^, 20^th^, 50^th^, 80^th^, and 95^th^ percentile.

**Fig. 10:**
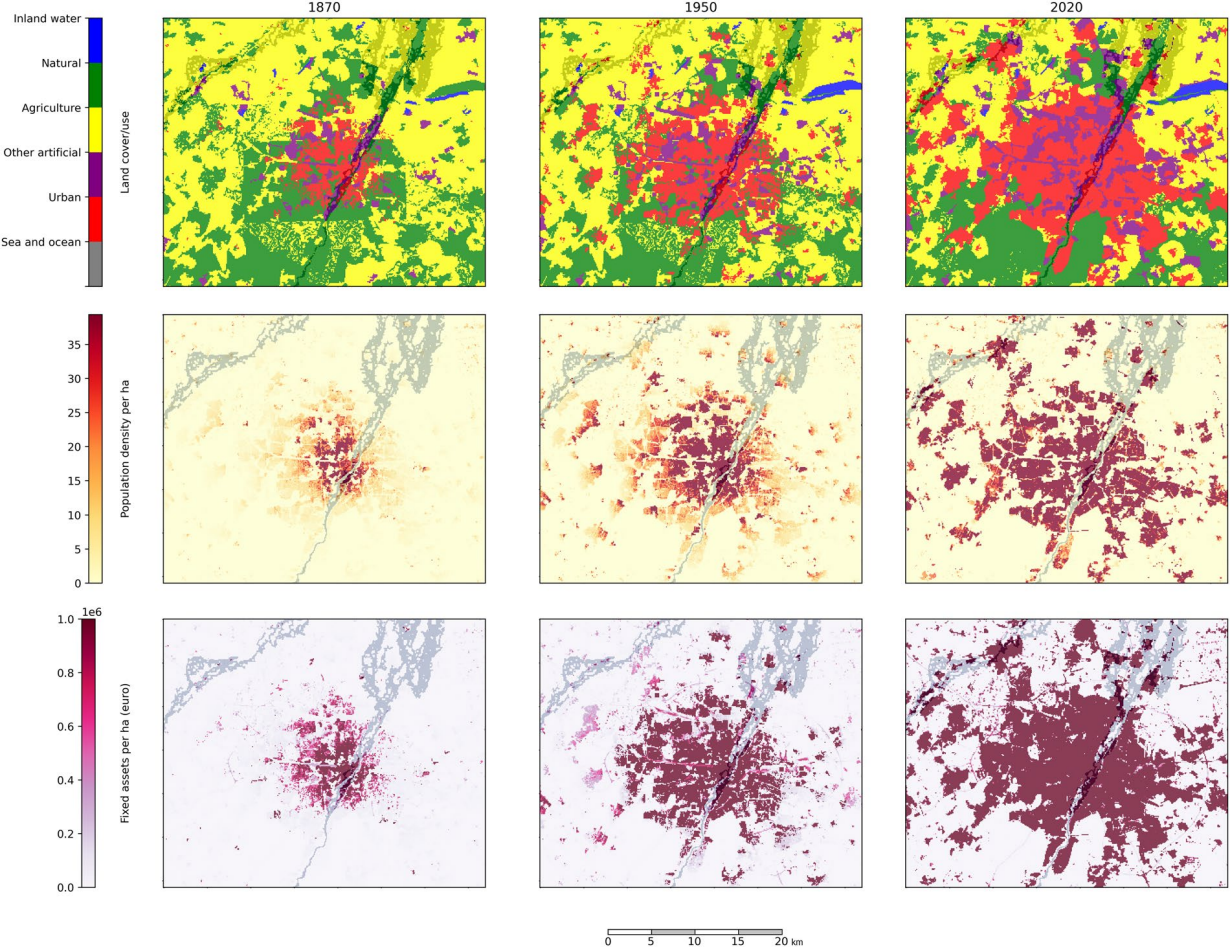
An example of modelled exposure growth in the vicinity of Munich (NUTS region DE212), southern Germany, between 1870 and 2020, contrasted with a 100-year river flood map (grey shading). Flood hazard zone from Alfieri *et al*.^57^.

The code also enables, for reproducibility, computing some of the input data. Many of the input datasets required extensive one-off preparations, hence only certain pre-processing steps could be included. Importantly, the population disaggregation routine described and validated in this study in can be rerun. The population thresholds for dasymetric mapping can also be recomputed, as well as the probability maps used in land-use modelling (step 10). Code for reproducing the validation of population change and land-use change is also included. Finally, the code enables visualising selected exposure information per flood event (from HANZE v1.0 database of past floods) in the form of graphs and maps. For any user-defined NUTS3 region, the code can generate an exposure map similar to Fig. 10.

Five illustrative examples of past flood events are shown in Table 15 to highlight how varied, and at times uncertain, exposure changes can be. They depend not only how far in the past the event has occurred (uncertainty increases with time), but also whether it occurred in the expansion zones of cities (where uncertainty is the highest) compared to a largely rural area, or what part of a NUTS 3 region is at risk (as the population or assets per region are defined by historical statistics, there would be no uncertainty if the entire region was a hazard zone). Maps of the affected areas, generated using the model’s code, are presented in Supplementary Fig. S10. In the future, we expect to use the model to attribute flood impacts to both environmental and economic drivers by linking HANZE more directly to climate and hydrological models^64,65^.

**Table 15:** Reported and normalized (exposure-adjusted) flood losses from large historical events.

Event	Reported losses at the time of the event				Normalized losses at 2020 level of exposure			
	Fatalities	Persons affected (‘000 s)	Economic loss (billions)		Fatalities	Persons affected (‘000 s)	Economic loss (billion euro)	
			Original currency	2020 euros			GDP-adjusted	FA-adjusted
Coastal flood in Hamburg, Germany, 1962	315	20.0	2.5 [Mark]	5.8	454 [344–464]	28.8 [21.9–29.5]	16.2 [15.1–16.3]	28.7 [26.4–28.8]
Riverine flood in southern France, 1930	230	16.2	1.0 [Franc]	0.8	582 [545–620]	41.0 [38.4–43.6]	10.4 [10.0–10.6]	7.8 [7.6–7.9]
Riverine flood in eastern Hungary, 1970	215	27.2	5.1 [Forint]	0.6	196 [193–200]	24.8 [24.4–25.4]	1.60 [1.58–1.62]	2.35 [2.33–2.37]
Riverine flood in north-eastern Italy, 1928	0	6.5	0.08 [Lira]	0.09	0	7.7 [7.2–8.5]	0.65 [0.64–0.67]	2.25 [2.19–2.34]
Coastal flood in Pärnu, Estonia, 2005	1	3.15	0.75 [Kroon]	0.09	1	3.29 [3.19–3.31]	0.09	0.10

Reported losses from HANZE v1.0^32^. Normalized losses are shown with 95% uncertainty intervals. FA: fixed assets.

The users should be aware of the limitations (e.g. not every land-use class is covered by the model, only the more important ones) and uncertainties (related to both modelling approach and quality of the input data). They were extensively discussed in relation to HANZE v1.0, therefore we refer the reader to Paprotny *et al*.^32^. In the future, we expect to use the model to attribute historical flood impacts to both environmental and economic drivers.

## Supplementary information

Supplementary Table S1

Supplementary Information

## Data Availability

The source code of HANZE v2.0 (implemented in Python 3.9) presented in the paper is archived at 10.5281/zenodo.7556953^63^. All necessary input data are archived at 10.5281/zenodo.6783023^62^. The flood impact data shown in Usage Notes, with a description of sources of the data, are available in the HANZE v1.0 repository^66^, 10.4121/collection:HANZE.
