# Supplementary material for: Population, land use and economic exposure estimates for Europe at 100 m resolution from 1870 to 2020
Source: Sci Data. 2023 Jun 8;10:372. doi: 10.1038/s41597-023-02282-0 (PMC10250532; doi:10.1038/s41597-023-02282-0)
Supplement: Supplementary file 2 — Supplementary Information [file 41597_2023_2282_MOESM2_ESM.docx]

Supplementary Information for “Population, land use and economic exposure estimates for Europe at 100 m resolution from 1870 to 2020”

Contents

[Supplementary Figure S1. HANZE domain 2](#_Toc136419073)

[Supplementary Figure S2. Dependency between average population density and the percentage of area covered 3](#_Toc136419074)

[Supplementary Figure S3. Regional data availability in HANZE 4](#_Toc136419075)

[Supplementary Figure S4. Zuiderzeewerken 5](#_Toc136419076)

[Supplementary Figure S5. VLAUs 6](#_Toc136419077)

[Supplementary Text S1. Local population estimates, 1961-2011 7](#_Toc136419078)

[Supplementary Text S2. Calibrating urban population change 9](#_Toc136419079)

[Supplementary Text S3. Data for the land-use transition model 11](#_Toc136419080)

[Supplementary Tables S3-S6. Input files 13](#_Toc136419081)

[Supplementary Table S7. Additional validation results. 16](#_Toc136419082)

[Supplementary Figure S9. Observed population change in Austria 17](#_Toc136419083)

[Supplementary Figure S10. Exposure to floods for selected flood events 18](#_Toc136419084)

## Supplementary Figure S1. HANZE domain


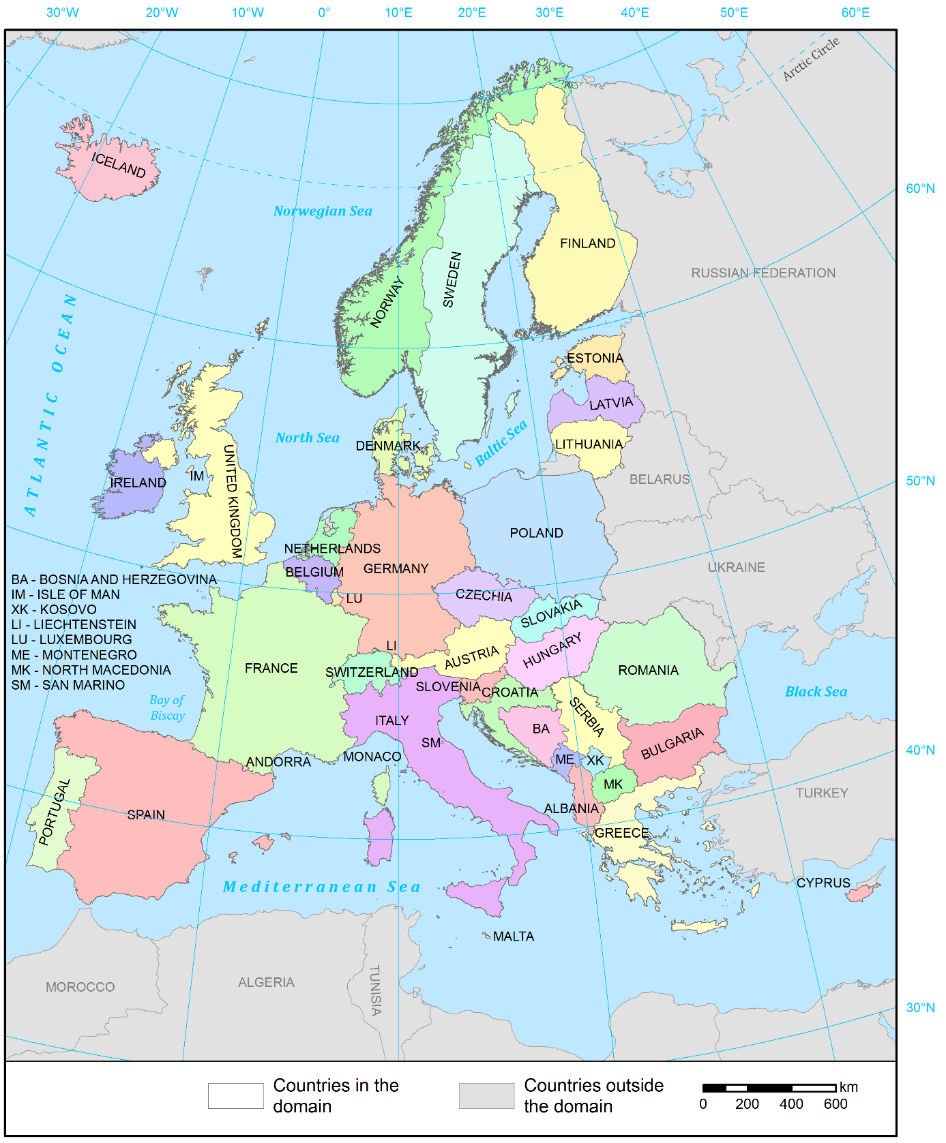


***Supplementary Figure S1.* HANZE domain. National boundaries outside the study area based on Natural Earth (**<https://www.naturalearthdata.com/downloads/10m-cultural-vectors/>**). See Table 5 in the main text for sources of boundary data within the study area.**

## Supplementary Figure S2. Dependency between average population density and the percentage of area covered


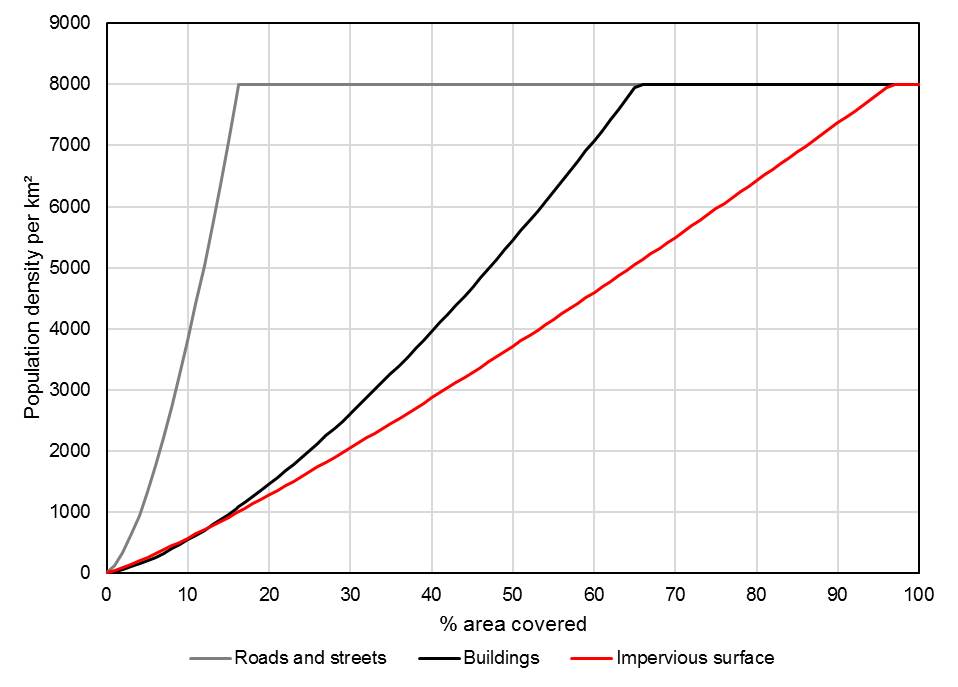


Supplementary Figure S2. Dependency between average population density and the percentage of area covered used for population disaggregation.

## Supplementary Figure S3. Regional data availability in HANZE


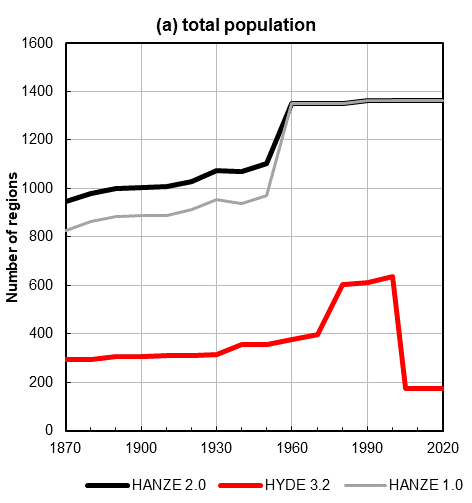

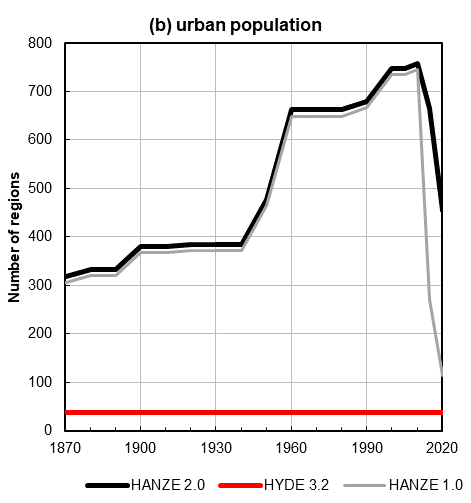

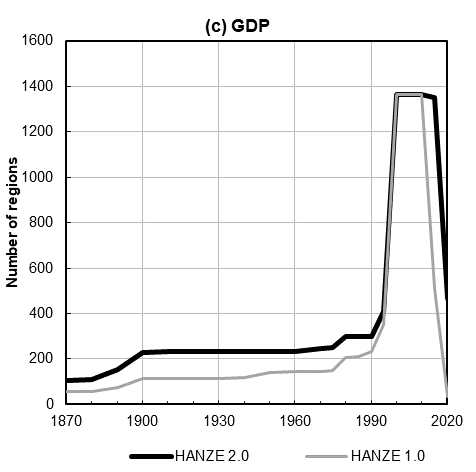


***Supplementary Figure S3.* Availability of regional data in HANZE (v1 from 2017 and v2 in this study) and HYDE v3.2. The data are for the domain of HANZE v1, i.e. without non-EU Balkan countries, and with a 10-yearly timestep for 1870-2000 and 5-yearly for 2000-2020, except GDP shown with a 10-yearly timestep for 1870-1970 and 5-yearly for 1970-2020.**

## Supplementary Figure S4. Zuiderzeewerken


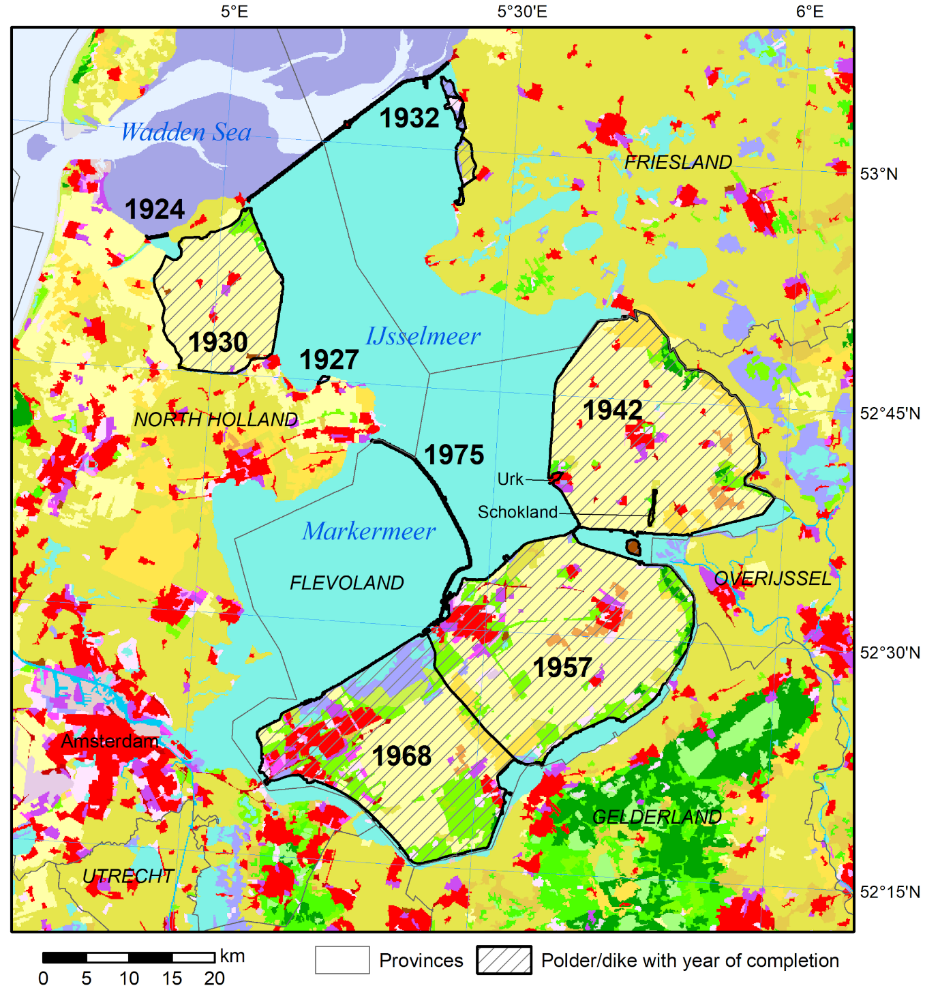


***Supplementary Figure S4. Zuiderzeewerken* land reclamations by year of construction, overlaying Corine Land Cover 2012 map (**[**https://land.copernicus.eu/pan-european/corine-land-cover/clc-2012**](https://land.copernicus.eu/pan-european/corine-land-cover/clc-2012)**).**

## Supplementary Figure S5. VLAUs


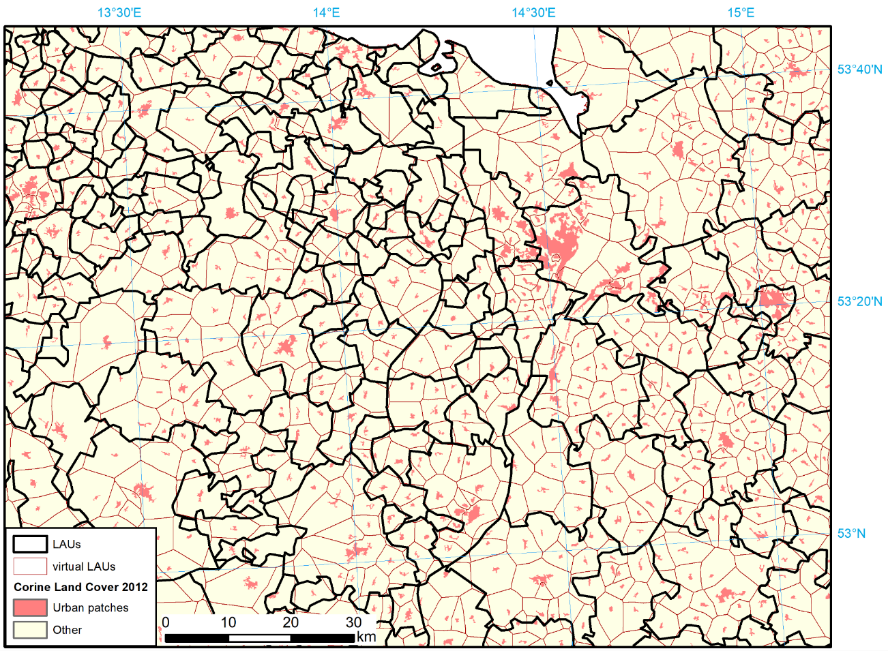


***Supplementary Figure S5. VLAUs (red lines) in a fragment of Germany and Poland compared with actual LAUs (black line) from Eurostat (2021).***

## Supplementary Text S1. Local population estimates, 1961-2011

Gløersen and Lüer (2013) collected population data at the level of local administrative units (LAUs) based on censuses made from ca. 1960 to 2011. The data were recalculated to a single set of subdivisions to allow consistent comparison through time. For most countries, data represent LAU level 2 (as defined at the time by Eurostat; currently one level of LAU is retained), which is the lowest level of administrative divisions in a given country. For Lithuania and Slovenia, a more aggregated level (LAU 1) was provided . The data was provided at census dates and interpolated (sometimes also extrapolated) to benchmark dates, spanning full decades from 1 January 1961 to 1 January 2011; the interpolated data were used in the analysis. The coverage of the study area is not complete. The following additions were made:

- In North Macedonia, there has been no census since 2002, hence mid-year estimates for 2011 from the Republic of North Macedonia State Statistical Office (<https://www.stat.gov.mk/OblastOpsto_en.aspx?id=2>) was used. Due to gaps in data and spatial data limitations, the population figures were aggregated to LAU 1 level, with 10 LAUs in the Skopje area aggregated further to a single unit;
- Data for Serbia was taken at LAU 1 level from the Statistical Office of the Republic of Serbia (<https://www.stat.gov.rs/en-us/oblasti/stanovnistvo/>);
- For five small countries and territories (Andorra, Isle of Man, Monaco, San Marino, the Vatican), population for the entire territory as compiled in the HANZE database was used.

The Eurostat’s dataset doesn’t also cover Albania, Bosnia and Herzegovina, Kosovo and Montenegro. Those countries were excluded from further analysis due to difficulty in gathering the necessary population and spatial data. Also, data is completely missing for 240 out of 614 LAUs in Cyprus, however the vast majority of those are located wholly or partially in Northern Cyprus, therefore outside this study’s domain. Data is also missing for some years in 15 LAUs located in Czechia (3), Cyprus (1), France (1), Germany (3), Malta (2) and Slovakia (5).

The tabular data were merged with spatial data from Eurostat. As the subdivisions refer to different time points for different countries, and are also not always internally consistent, several datasets produced by EuroGeographics and provided to Eurostat were used (“Communes” for different years and “Census units 2011”). Due to the resolution of spatial data, population figures for Denmark, Greece, North Macedonia and Portugal had to be aggregated to LAU 1 level. For some countries, individual local units had to be aggregated, split or redrawn in order to match the subdivisions used in the population dataset. For 37 out of 3441 LAUs in Ireland and 86 out of 9499 LAUs in the United Kingdom it was to possible to match tabular and spatial data due to large administrative boundary changes.

The final dataset contains 109,419 LAUs (Fig. S6). Of these, 378 LAUs are not usable due to missing population data and 364 LAUs have no population^^[[1]](#footnote-1)^^, resulting in a total of 108,679 LAUs with generally consistent population data for 1961-2011. It was noticed for a small number of cases (e.g. for some locations in Italy) that the data were not fully adjusted for administrative boundary changes. Still, the benefit of the dataset for analysing for population changes in Europe is very large.


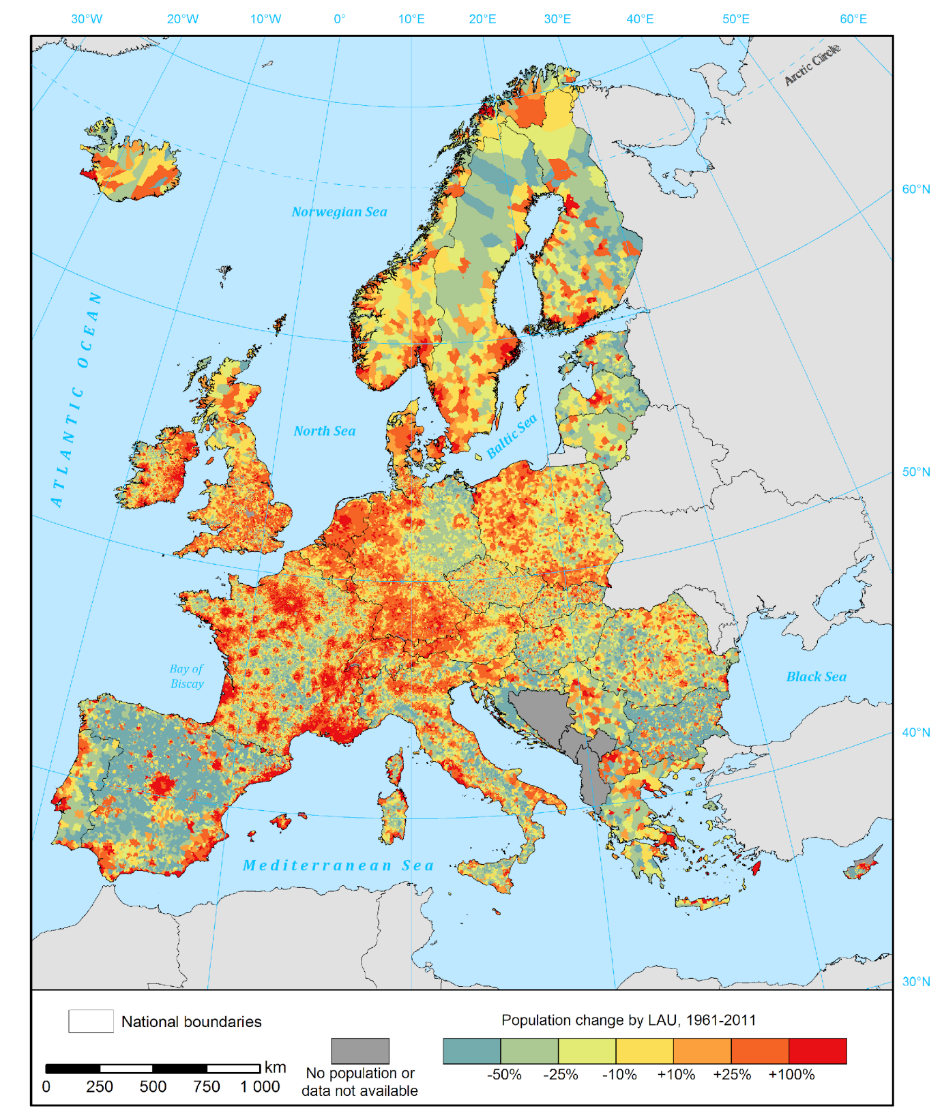


***Supplementary Figure S6. Population change by local administrative unit between 1961 and 2011 (see text for data sources).* National boundaries outside the study area based on Natural Earth (2022).**

## Supplementary Text S2. Calibrating urban population change

The calibration utilizes Clark’s (1951) model of population density, which he described with an exponential function:

$$y=Ae^{-bx} (1)$$

where *y* is the population density (in persons per ha), *x* is the distance from the city centre (in km), *A* and *b* are exponential function coefficients. Clark (1951, 1967) provided estimates of *A* and *b* for 16 cities in 9 countries for 29 time points. Hourihan (1982) provided additional estimates for 3 cities from several censuses, of which 13 cases were used (estimates made with only a few data points were excluded). That gives a total of 42 estimates spanning a whole century, from 1871 to 1971 (Table S2). In the population dataset constructed here the population density was calculated for 500 m wide zones around (arbitrarily chosen) city centre, interpolated to match the time points from literature and then fitted to an exponential function. The model was run many times using random weighting of five datasets indicating distance from urban centre. A comparison of function parameters for a most optimal combination is presented in Fig. S7. Overall, the fit is moderate, but a better match of modelled and observed estimates of eq. 1 parameters would be difficult, since the exponential curve fits are very sensitive to the sample size, i.e. spatial resolution of data and maximum distance from the city centre. The latter is not known for all cities. Additionally, the source literature studies used census wards of different sizes instead of a disaggregated population grid used here.


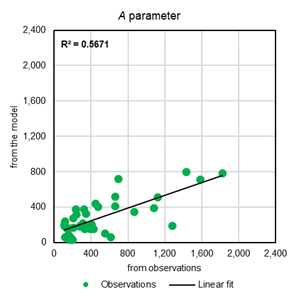

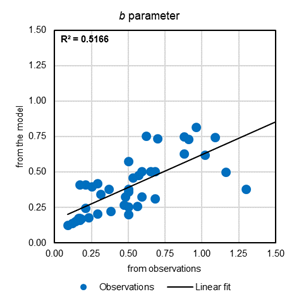


***Supplementary Figure S7. Estimates of* A *and* b *parameters (eq. A1) from modelled and observed population data.***

***Supplementary Table S2. Estimates of urban population density. A, b – exponential function parameters (adjusted to give population density in persons per ha, rather than persons per sq. mile as in Clark 1951 and Hourihan 1982). D – maximum distance from the city centre (km), for which population data were used to calculate exponential function parameters (values in red are estimates, as the source does not specify the distance).***

| **Name** | **Region** | **Year** | **A** | **b** | **D** | **Source** |
| --- | --- | --- | --- | --- | --- | --- |
| Aarhus | DK042 | 1950 | 279 | 0,96 | 5 | Clark 1967 |
| Berlin | DE300 | 1885 | 1120 | 0,68 | 8 | Clark 1951; Clark 1967 |
| Berlin | DE300 | 1900 | 1580 | 0,59 | 10 | Clark 1951; Clark 1967 |
| Birmingham | UKG31 | 1921 | 401 | 0,50 | 11 | Clark 1967 |
| Birmingham | UKG31 | 1938 | 201 | 0,29 | 12 | Clark 1967 |
| Budapest | HU101 | 1935 | 1080 | 0,56 | 5 | Clark 1951; Clark 1967 |
| Copenhagen | DK011 | 1940 | 231 | 0,37 | 7 | Clark 1967 |
| Cork | IE025 | 1926 | 199 | 1,02 | 3 | Hourihan 1982 |
| Cork | IE025 | 1936 | 177 | 0,88 | 3 | Hourihan 1982 |
| Cork | IE025 | 1951 | 176 | 0,91 | 4 | Hourihan 1982 |
| Cork | IE025 | 1961 | 114 | 0,70 | 4 | Hourihan 1982 |
| Cork | IE025 | 1971 | 158 | 0,62 | 4 | Hourihan 1982 |
| Dublin | IE021 | 1901 | 391 | 0,68 | 4 | Hourihan 1982 |
| Dublin | IE021 | 1911 | 379 | 0,65 | 4 | Hourihan 1982 |
| Dublin | IE021 | 1926 | 352 | 0,59 | 4 | Hourihan 1982 |
| Dublin | IE021 | 1936 | 270 | 0,53 | 6 | Clark 1951; Clark 1967 |
| Dublin | IE021 | 1951 | 106 | 0,25 | 8 | Hourihan 1982 |
| Dublin | IE021 | 1961 | 105 | 0,21 | 8 | Hourihan 1982 |
| Dublin | IE021 | 1971 | 113 | 0,17 | 8 | Hourihan 1982 |
| Frankfurt am Main | DE712 | 1890 | 550 | 1,16 | 5 | Clark 1967 |
| Frankfurt am Main | DE712 | 1933 | 340 | 0,57 | 6 | Clark 1967 |
| Leeds | UKE42 | 1951 | 116 | 0,31 | 9 | Clark 1967 |
| Limerick | IE023 | 1961 | 136 | 1,09 | 3 | Hourihan 1982 |
| Limerick | IE023 | 1971 | 126 | 0,88 | 3 | Hourihan 1982 |
| Liverpool | UKD72 | 1921 | 1275 | 0,50 | 9 | Clark 1951; Clark 1967 |
| London | UKI11 | 1871 | 865 | 0,38 | 17 | Clark 1967 |
| London | UKI11 | 1901 | 660 | 0,23 | 20 | Clark 1967 |
| London | UKI11 | 1921 | 443 | 0,17 | 25 | Clark 1967 |
| London | UKI11 | 1931 | 475 | 0,17 | 26 | Clark 1967 |
| London | UKI11 | 1939 | 320 | 0,14 | 28 | Clark 1967 |
| London | UKI11 | 1951 | 240 | 0,12 | 29 | Clark 1967 |
| London | UKI11 | 1961 | 205 | 0,09 | 33 | Clark 1967 |
| Manchester | UKD31 | 1931 | 155 | 0,16 | 18 | Clark 1951 |
| Manchester | UKD31 | 1939 | 143 | 0,18 | 20 | Clark 1967 |
| Oslo | NO011 | 1938 | 308 | 0,50 | 4 | Clark 1951; Clark 1967 |
| Paris | FR101 | 1896 | 1430 | 0,50 | 12 | Clark 1951; Clark 1967 |
| Paris | FR101 | 1931 | 1820 | 0,47 | 14 | Clark 1951; Clark 1967 |
| Paris | FR101 | 1946 | 695 | 0,21 | 16 | Clark 1967 |
| Stockholm | SE110 | 1880 | 610 | 1,30 | 10 | Clark 1967 |
| Stockholm | SE110 | 1940 | 425 | 0,48 | 10 | Clark 1967 |
| Vienna | AT130 | 1890 | 660 | 0,50 | 7 | Clark 1951; Clark 1967 |
| Zurich | CH040 | 1936 | 328 | 0,29 | 8 | Clark 1967 |

## Supplementary Text S3. Data for the land-use transition model

Data for the land-use transition model, implemented as Bayesian Network, was obtained by sampling the CLC inventory. Firstly, vector layers of CLC-Changes 2000-2006, 2006-2012 and 2012-2018 were obtained (1,194,980 patches). The CLC classes before and after transition were grouped together as follows:

- Urban fabric (CLC 111-112);
- Other artificial (CLC 121-142);
- Croplands (CLC 211-223 and 241-244);
- Pastures (CLC 231);
- Natural (CLC 311-324, 333 and 411-412);
- Other (CLC 331-332, 334-335, 421-523).

CLC-Changes patches transitioning within a given group, or transitioning from/to “Other” classes were excluded from further analysis, leaving 240,870 patches of varying size. To sample the inventory, a “fishnet” of 25x25 km cells was created and clipped to the land mask of the baseline land cover/use dataset. It was further clipped to the remaining CLC-Changes patches. In each cell of the fishnet, $1+\frac{A}{7}$ samples (rounded to the nearest integer) within the borders of CLC-Changes patches were generated^^[[2]](#footnote-2)^^, where $A$ is the area of all patches within a fishnet cell (in hectares). The samples were generated at least 100 meters apart due to the resolution of most raster data used throughout this study. In total, 513,915 locations with land-use transitions were obtained. Then, an equal number of samples in each fishnet cell were generated in the remaining area of the cells. In this way, the same number of random locations where no land-use transitions took place between 2000 and 2018 was obtained with the same spatial distribution as the other dataset. Instances of transitions are many times fewer in reality, but as we are interested in the relative probability of transition between CLC classes rather than the total probability, we can sample a greater proportion of transitions to better quantify the patterns of land-use changes. An example of sample locations is presented in Fig. S12.

A smaller validation dataset was created as well. A new set of random points was generated in the fishnet, but the number of samples per cell was capped at 15, resulting in 97,790 samples each for transitions and non-transitions, with a much more even spatial distribution throughout Europe.

The sample locations were used to extract data from various raster datasets, as follows:

- Population density in 2011 computed using kernel density and per VLAU
- Euclidean distances from urban centres based on five datasets
- Elevation (in meters) and slope (per mille) computed from EU-DEM dataset at 100 m resolution (<https://ec.europa.eu/eurostat/web/gisco/geodata/reference-data/elevation/eu-dem/eu-dem-laea>);
- Agricultural suitability indices calculated by FAO in the Global Agro-Ecological Zoning version 4 (<https://gaez-data-portal-hqfao.hub.arcgis.com/>) database for five different crops (alfalfa, grass, wheat, rye and white potato):
  - Suitability index range (0–10000), with all land in grid cell under rainfed conditions;
  - Output density (potential production divided by total grid cell area) under rainfed conditions;
  - Agro-climatic potential yield with an available water content of 200 mm/m (under irrigation conditions).

The agricultural indices were all for the historical period 1971-2000 using CRUTS32 climate data, assuming high input level and without CO_2_ fertilization. The indices combine data on climate, soil and terrain to estimate potential yield of various crops. The resolution of this dataset is 5’ (about 4–7 km, depending on location).


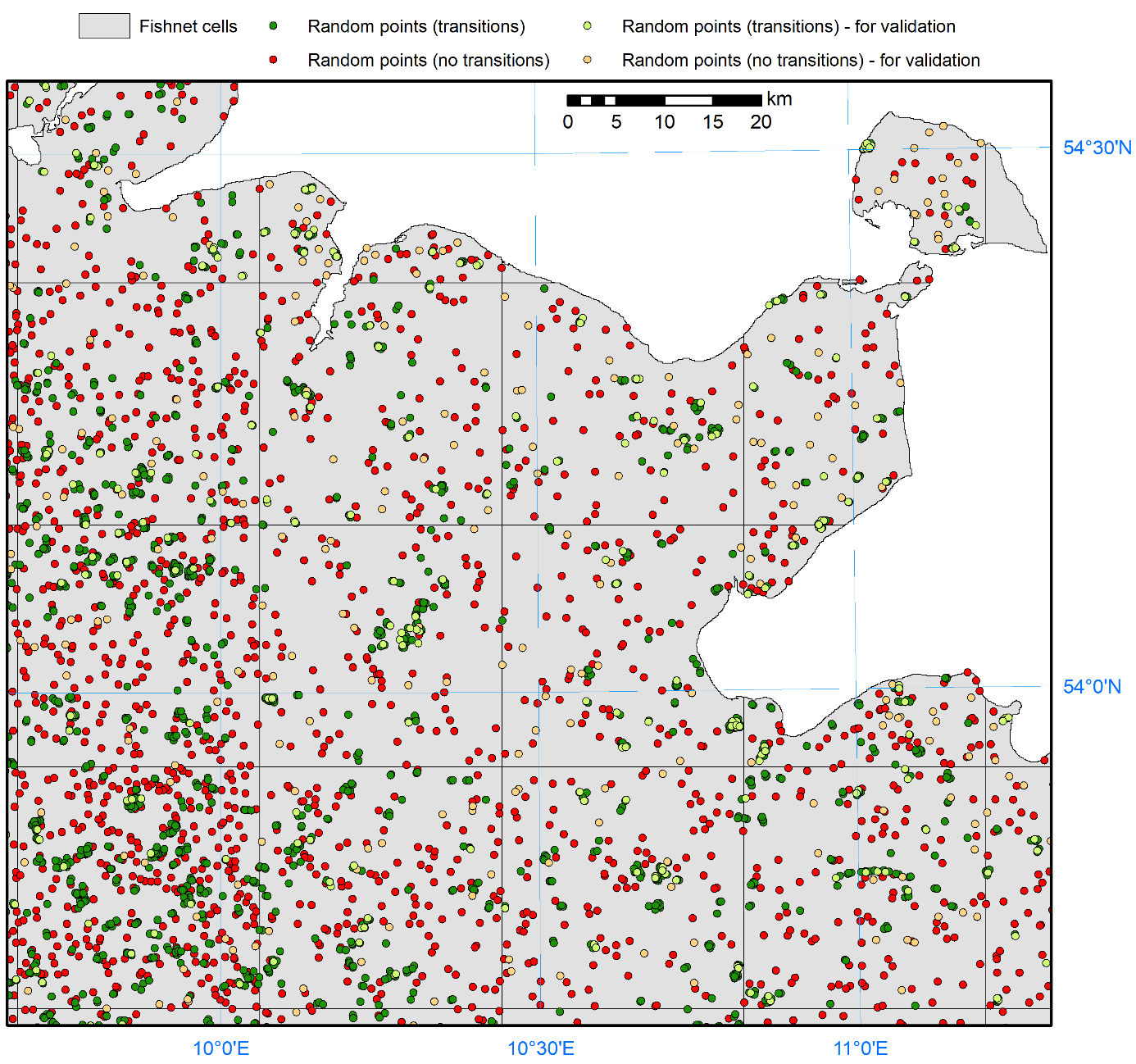


***Supplementary Figure S8. Example of sample locations generated for a fragment of northern Germany.***

## Supplementary Tables S3-S6. Input files

***Supplementary Table S3.* List of main input files of HANZE v2.0 model.**

| **File** | **Format** | **Variables / contents** |
| --- | --- | --- |
| **Input** |  |  |
| Region_database_population_lu | Excel file | Input land use/cover and population data |
| Region_database_economy | Excel file | Input and auxiliary economic data |
| CLC_base_HANZE2 | 8-bit GeoTIFF | Baseline land cover/use type, 44 classes according to Corine Land Cover |
| Population_100m | 16-bit GeoTIFF | Total baseline (disaggregated) population per 100 m grid cell (in persons) |
| IMD2012_extent_adjusted | 8-bit GeoTIFF | Soil sealing degree in % |
| NUTS2010_final_100m | Shapefile | NUTS3 (version 2010) region definitions |
| NUTS2010_100m_c | 8-bit GeoTIFF | NUTS3 region definitions (numerical value from vector file attribute table) |
| gras200a_yld_LAEA2 | 16-bit GeoTIFF | Agro-climatic potential yield for grass |
| ylHr0_whe_LAEA2 | 16-bit GeoTIFF | Output density for wheat |
| Airports_year_v2 | 8-bit GeoTIFF | Airports by year of construction |
| Slope_per_mille_int_masked | 16-bit GeoTIFF | Slope (per mille) from EU-DEM |
| ESM2012_street_ext_adj | 8-bit GeoTIFF | Surface covered by roads and streets (%) from European Settlement Map 2012 |
| Industry_centroids_int | 32-bit GeoTIFF | Distance from centroids of industrial CLC patches in meters |
| NL_polders | 16-bit GeoTIFF | Year of construction of Dutch polders |
| CLC_141_142_selected_v2 | 32-bit GeoTIFF | Green urban areas and sport facilities than are adjacent to selected artificial surfaces |
| CLC2012_urban_d_int | 32-bit GeoTIFF | Euclidean distance from centroids of urban CLC 2012 patches |
| Clusters2011_high_density_d_int | 32-bit GeoTIFF | Euclidean distance from centroids of high-density population clusters |
| KernelDensityPop1km_int | 32-bit GeoTIFF | Kernel population density with 10-km radius |
| UN_agglomerations_d_int | 32-bit GeoTIFF | Euclidean distance from centres of large agglomerations and capital cities |
| UrbanAudit2018_d_int | 32-bit GeoTIFF | Euclidean distance from centroids of cities in Urban Atlas 2018 |
| VirtualLAU_PD_int_new | 32-bit GeoTIFF | Population density of Virtual LAUs (VLAUs) |
| LAU_data | CSV | Population data (1961-2011) per LAU |

***Supplementary Table S4. List of input probability maps from the Bayesian Network model.***

| BN_to_urban | 16-bit GeoTIFF | Probability map of transition from non-urban to urban after the baseline year |
| --- | --- | --- |
| BN_to_crop | 16-bit GeoTIFF | Probability map of transition from non-cropland to cropland after the baseline year |
| BN_to_past | 16-bit GeoTIFF | Probability map of transition from non-pasture to pasture after the baseline year |
| BN_from_crop | 16-bit GeoTIFF | Probability map of transition from cropland to non-cropland after the baseline year |
| BN_from_past | 16-bit GeoTIFF | Probability map of transition from pasture to non-pasture after the baseline year |
| BN_to_crop_p | 16-bit GeoTIFF | Probability map of transition from non-cropland to cropland before the baseline year |
| BN_to_past_p | 16-bit GeoTIFF | Probability map of transition from non-pasture to pasture before the baseline year |
| BN_from_crop_p | 16-bit GeoTIFF | Probability map of transition from cropland to non-cropland before the baseline year |
| BN_from_past_p | 16-bit GeoTIFF | Probability map of transition from pasture to non-pasture before the baseline year |

***Supplementary Table S5. List of pre-processing data (for reproduction of certain inputs)***

| Pure_Population_CLC_cells | CSV | GEOSTAT population 1 km grid cells covered with a single CLC class |
| --- | --- | --- |
| Population_thresholds | CSV | Thresholds for population disaggregation |
| ESM2012_buildings | 8-bit GeoTIFF | Surface covered by buildings (%) from European Settlement Map 2012 |
| GEOSTAT_extent_adjusted | 8-bit GeoTIFF | GEOSTAT 1 km population grid |
| ESM_GEOSTAT_statistics | CSV | Average population per surface covered by buildings (%) |
| ESM_Street_GEOSTAT_statistics | CSV | Average population per surface covered by roads and streets (%) |
| IMP_GEOSTAT_statistics | CSV | Average population per surface covered by impervious surfaces (%) |
| CLC_changes_sample_data | CSV | CLC land-use transition samples used to train the BN land-use model |
| CLC_changes_nosample_data | CSV | CLC land-use non-transition samples used to train the BN land-use model |
| BN_sample_data | NumPy file | Processed CLC land-use transition and non-transition samples used to train the BN land-use model |

***Supplementary Table S6. List of validation and analysis datasets.***

| LAU2_Austria_pop | Shapefile | Population of Austria by municipality, 1870-2020, for validation |
| --- | --- | --- |
| BN_sample_data_validation | NumPy file | CLC land-use transition and non-transition samples used to validate the BN land-use model |
| RAIN_coastalmap_100y | 8-bit GeoTIFF | Coastal flood hazard map, 100-year return period |
| JRC_flood_mask_100 | 8-bit GeoTIFF | River flood hazard map, 100-year return period |
| Flood_events_v1.0_list | Excel file | Data on damaging floods, 1870-2016, from HANZE v1.0 |

## Supplementary Table S7. Additional validation results.

***Supplementary Table S6*. Correctly identified transitions of land use in the validation dataset.**

| **LAU population class** | **Number of LAUs (% share)** | **Population in LAUs (% share)** | **Average relative error (%) -**  **HANZE** | **Average relative error (%) -**  **HYDE** |
| --- | --- | --- | --- | --- |
| **Europe, 1960** |  |  |  |  |
| <1000 persons | 53% | 6% | 46% | 58% |
| 1000–9999 persons | 40% | 34% | 32% | 39% |
| 10000 and more | 7% | 60% | 20% | 30% |
| **Austria, 1870** |  |  |  |  |
| <1000 persons | 36% | 10% | 54% | 52% |
| 1000–2499 persons | 47% | 35% | 31% | 36% |
| 2500 and more | 17% | 55% | 41% | 57% |

## Supplementary Figure S9. Observed population change in Austria


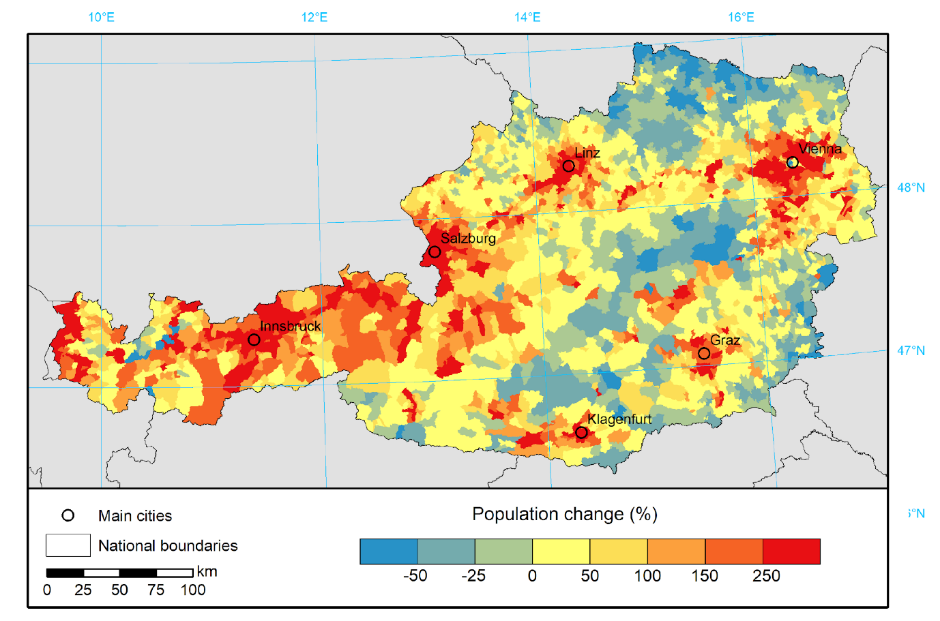


***Supplementary Figure S9*. Observed population change in Austria by local administrative unit, 1870-2020 (based on Statistik Austria,** <http://www.statistik.at/>**).**

## Supplementary Figure S10. Exposure to floods for selected flood events

**
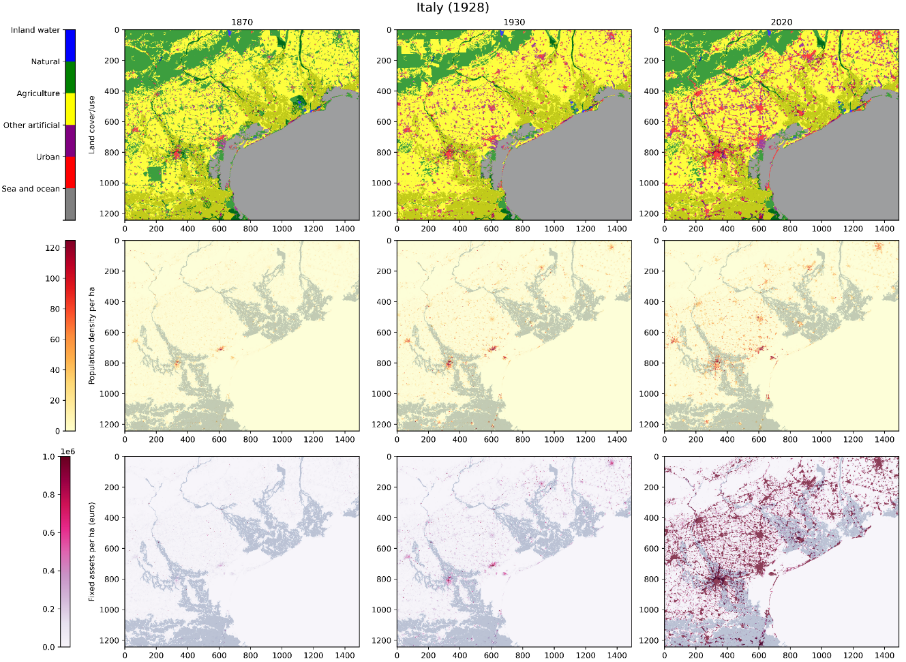
**


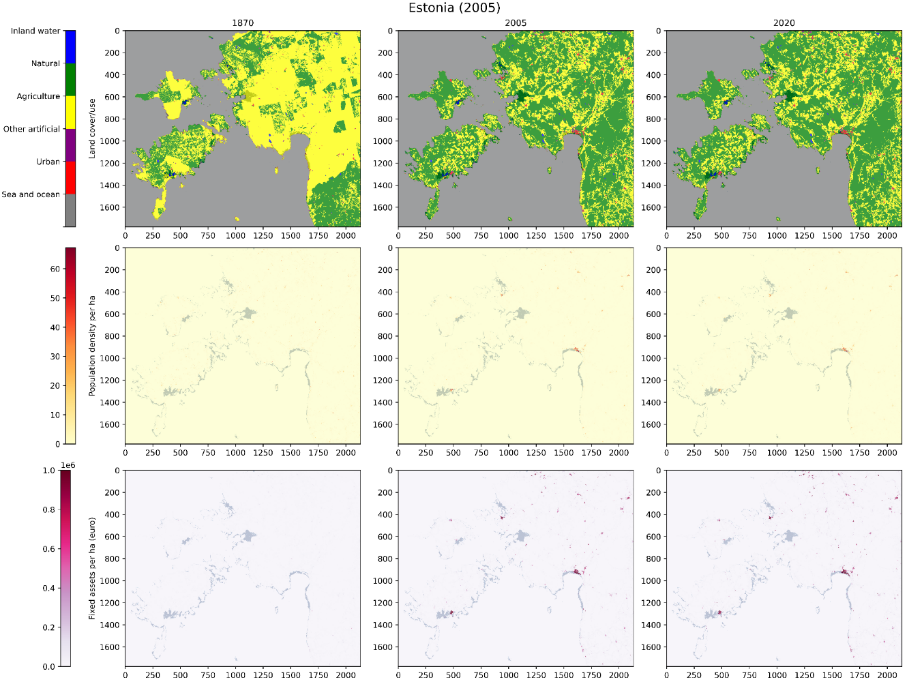


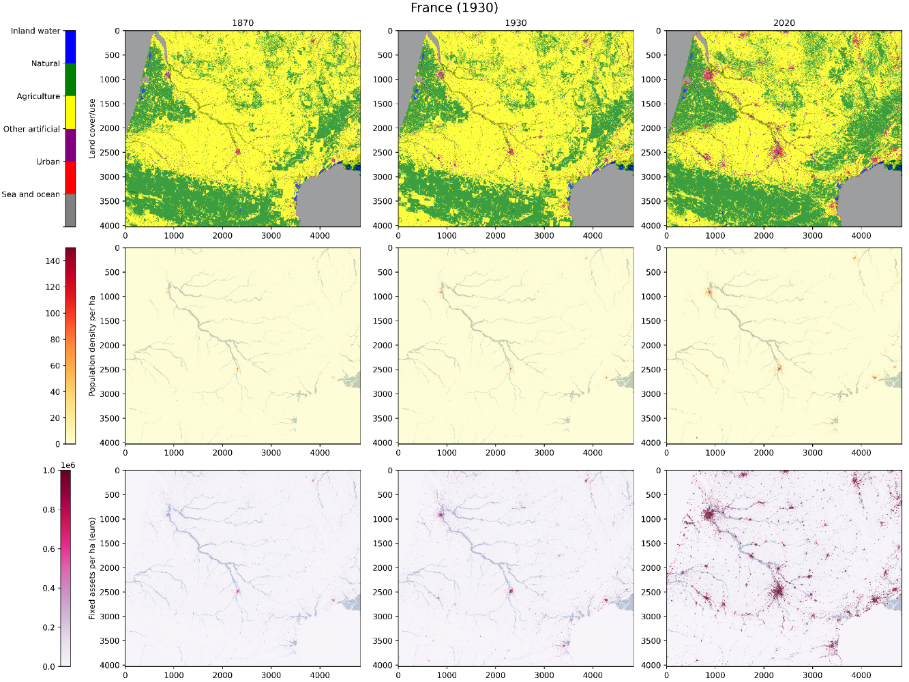


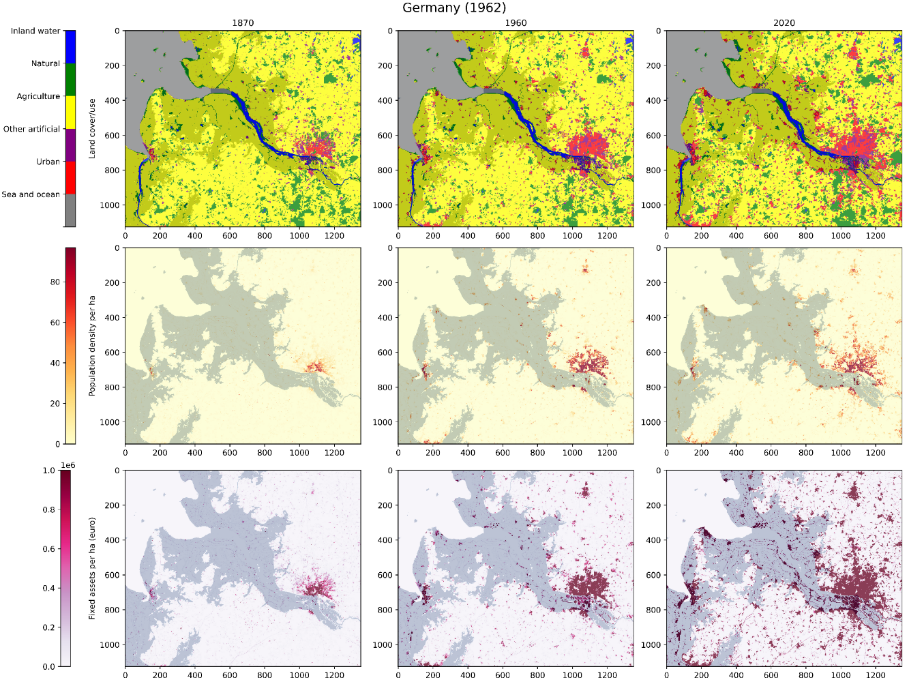


***
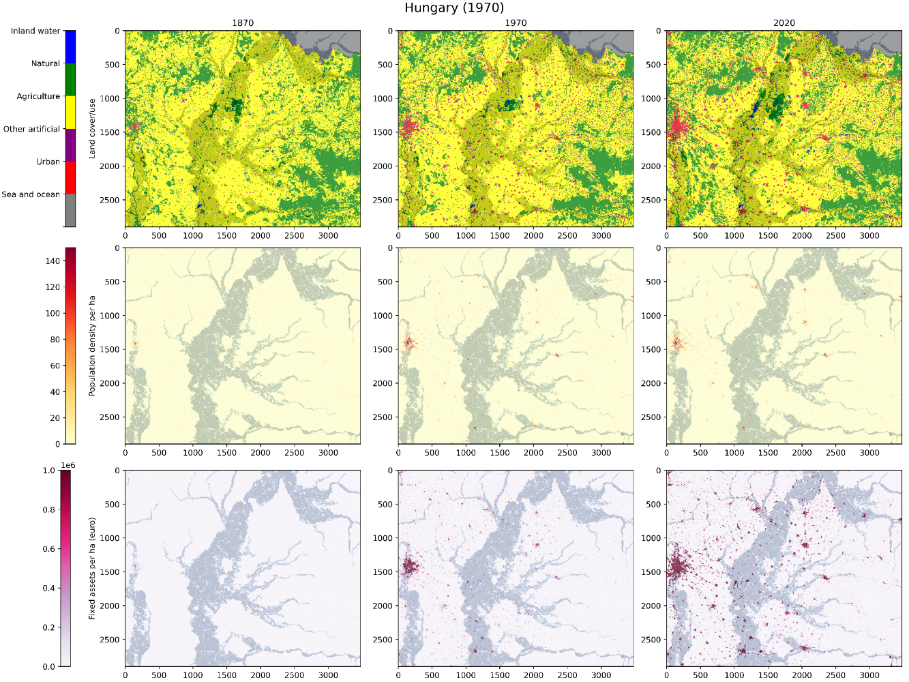
***

***Supplementary Figure S10. Exposure in the vicinity of floods described in the paper, in 1870, timestep nearest to the year of the event and 2020. River or coastal flood hazard zone is shown in the background.***

1. These are non-municipal areas, e.g. lakes, forests or military bases, occurring in Cyprus, Estonia, France, Germany, Lithuania, North Macedonia, Spain and Switzerland. [↑](#footnote-ref-1)
2. Using „Create Random Points” tool in ArcGIS 10.7.1 [↑](#footnote-ref-2)
